# Transition from Ginseng Root Rot Disease-Conducive Soil to -Suppressive Soil Mediated by *Pseudomonadaceae*

**DOI:** 10.1128/spectrum.01150-23

**Published:** 2023-07-05

**Authors:** Gyeongjun Cho, Da-Ran Kim, Youn-Sig Kwak

**Affiliations:** a Division of Agricultural Microbiology, Department of Agricultural Biology, National Institute of Agriculture Science, Rural Development Administration, Wanju, Republic of Korea; b Division of Applied Life Science and RILS, Gyeongsang National University, Jinju, Republic of Korea; Yeungnam University

**Keywords:** conducive soil, microbiota community, nitrogen fixation, suppressive soil, *Panax ginseng*, root rot disease

## Abstract

Ginseng is a popular medicinal herb with established therapeutic effects such as cardiovascular disease prevention, anticancer effects, and anti-inflammatory effects. However, the slow growth of ginseng due to soilborne pathogens has been a challenge for establishing new plantations. In this study, we investigated root rot disease associated with the microbiota in a ginseng monoculture model system. Our results showed that a collapse of the early microbiota community inhibiting root rot disease was observed before the disease became severe, and nitrogen fixation was necessary to support the initial microbiota community structure. Furthermore, changes in the nitrogen composition were essential for the suppression of pathogen activity in early monoculture soils. We hypothesize that *Pseudomonadaceae*, a population built up by aspartic acid, can inhibit the occurrence of root rot disease in ginseng and that specific management practices that maintain a healthy microbiome can be implemented to prevent and mitigate the disease. Our findings provide insights into the potential use of specific members of the microbiota for controlling root rot disease in ginseng cultivation.

**IMPORTANCE** Understanding the initial soil microbiota and community shifts in a monoculture system is critical for developing disease-suppressive soils for crop production. The lack of resistance genes against soilborne pathogens in plants highlights the need for effective management strategies. Our investigation of root rot disease and initial microbiota community shifts in a ginseng monoculture model system provides valuable insight into the development of conducive soil into specific suppressive soil. With a thorough understanding of the microbiota in disease-conducive soil, we can work toward the development of disease-suppressive soil to prevent outbreaks and ensure sustainable crop production.

## INTRODUCTION

Panax ginseng C. A. Meyer, commonly known as ginseng, is a medicinal plant indigenous to Northeast Asia. The cultivation of ginseng began due to its recognized medicinal properties in oriental medicine. In recent years, numerous studies have been conducted to validate its medicinal effects, including its hepatoprotective effect (1), heart-protective effect (2), anticancer effect (3), and anti-inflammatory effect (4). Consequently, ginseng and ginseng-based processed foods have gained popularity worldwide as health supplements (5). However, the cultivation of ginseng can be challenging due to its slow growth, making it susceptible to soilborne diseases, which are prevalent in fields where ginseng has previously been grown. This makes farmers reluctant to recultivate ginseng in such fields. In the Republic of Korea, root rot, which is a major soilborne disease, can cause a decline in quality and the disappearance of ginseng seedlings due to rotting without a trace. Yield losses of up to 30 to 60% have been reported. Causal pathogens of root rot disease in ginseng include Cylindrocarpon destructans (6) and Fusarium spp. (7, 8), which are predominantly comprised of F. solani, F. oxysporum, and F. moniliforme (9, 10).

Plant pathogens are a major cause of crop loss worldwide, with necrotrophic or heminecrotrophic fungal pathogens being particularly devastating. Plants lack natural resistance genes against these pathogens, and the prevalence of monoculture farming practices can exacerbate disease severity (11). Crop monoculture, especially in the early stages, can result in the enhancement of disease severity in the soil, a phenomenon referred to as conducive soil. This is because of the lack of genetic diversity, which makes crops more vulnerable to disease. In contrast, suppressive soil is characterized by a disease decline that occurs after a severe outbreak (12). It is believed that beneficial microorganisms in suppressive soil play a critical role in protecting plants from soilborne diseases. Suppressive soil is classified into two types: general and specific (13). General suppressive soil has the ability to inhibit soilborne pathogen growth or activity, attributed to the collective competition and antagonistic activity of various bacteria against the pathogen (14). In contrast, specific suppressive soil is characterized by a disease-reducing ability that is derived from one or several specific microbial species (11, 12, 15). Compared to general suppression, specific suppression is easy to transplant because only a few effective mechanisms need to be reproduced. This is due to the fact that specific suppressive soil is derived from one or a few specific microbial species, while general suppression results from the collective activity of various microbes in the rhizosphere.

The rhizosphere, originally defined by Hiltner (16) as the zone of soil around plant roots where microbial activity occurs, has been redefined by Čatská et al. (17) as the area influenced by plant root exudates and colonized by rhizobacteria. Interactions between bacteria and plant roots in the rhizosphere play a critical role in promoting plant growth, nutrition, and quality through carbon sequestration, ecosystem functioning, and nutrient cycling (18). Among these interactions, plant-growth-promoting rhizobacteria (PGPR) have a particularly positive effect, either directly or indirectly. *Bacillus* spp. and Pseudomonas spp. are well-known examples of PGPR that have been shown to trigger induced systemic resistance (ISR), and they are being studied as potential biocontrol agents and biofertilizers (13, 18).

Cho (19) investigated the ginseng monoculture cultivation model, which used inherited soil, and showed the collapse of the early microbiota community structure leading to the influence of seven taxa, *Beijerinckiaceae*, *Comamonadaceae*, *Devosiaceae*, *Rhizobiaceae*, *Sphingomonadaceae*, *Sphingobacteriaceae*, and *Xanthomonadaceae* (19). These influencer taxa collectively suppressed ginseng root rot disease, regardless of the population density of the root rot pathogen F. solani. Most of these influencer taxa were found to have a significant relationship with nitrogen metabolism capabilities and led the way in nitrogen fixation within the early microbiota community of the rhizosphere. Based on those findings, we hypothesized that the amino acids circulating after nitrogen fixation and the nitrogen composition would have a significant impact on the early microbiota community. To better understand the importance of nitrogen sources in the evolution of soil from conducive to suppressive, this study was planned to follow up on that previous research (19). This study used bulk soil inherited from a ginseng monoculture (10th cycle) and employed ginseng seedlings to examine the changes in the microbiota community after ammonium (NH_4_Cl) treatment resulting from nitrogen fixation. Also, glutamic acid (Glu), aspartic acid (Asp), and asparagine (Asn) were used because they are directly biosynthesized from ammonium, and valine (Val) was used as a control because it is made in a synthesis process that is distantly related to that of ammonium (20). Overall, this study aims to expand and further investigate the impact of nitrogen fixation on the microbiota community structure and its implications for soil evolution.

## RESULTS

### Effect of the nitrogen source on ginseng root rot and growth.

**FIG 1:**
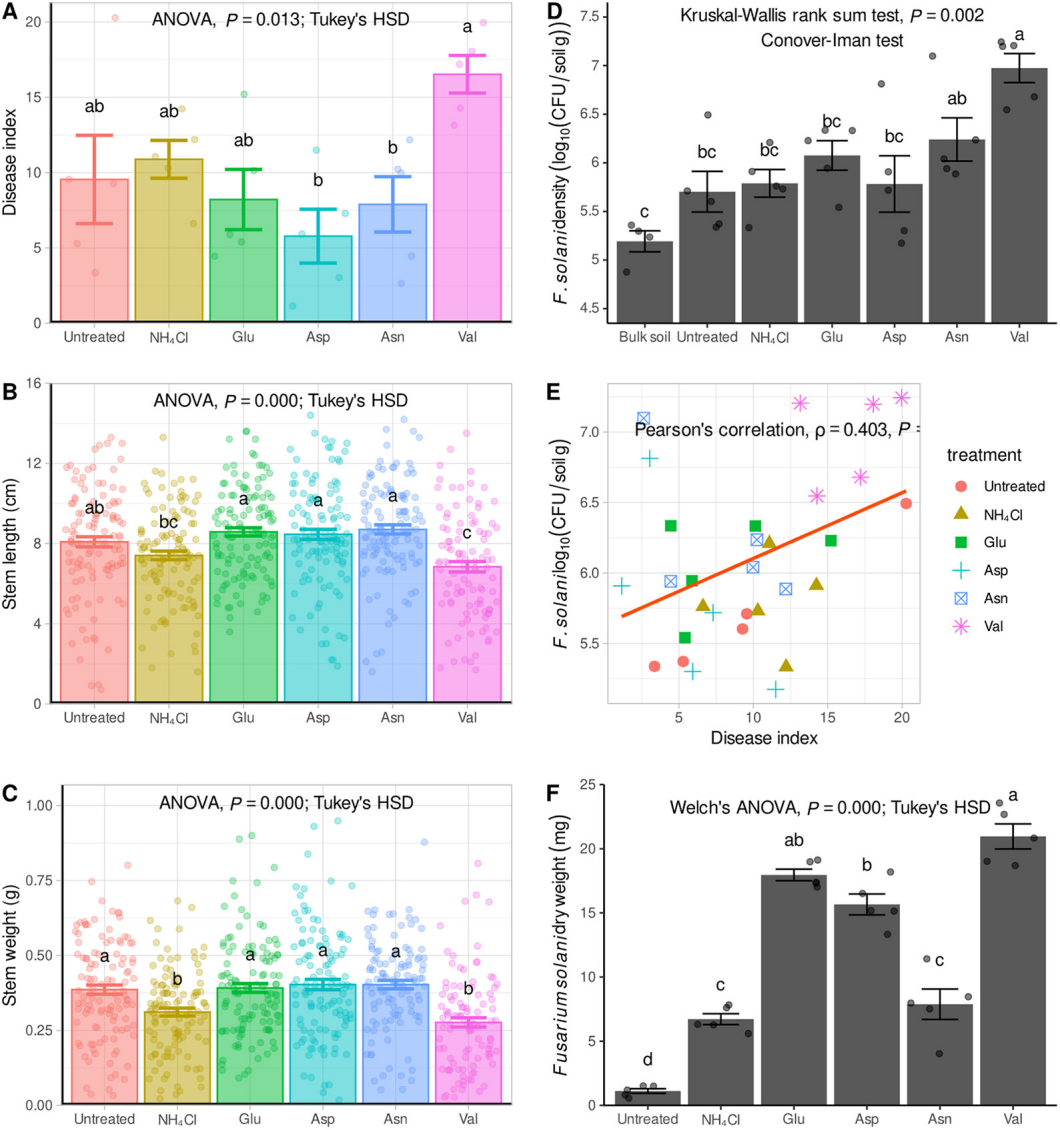
Effects of different nitrogen sources on the ginseng phenotype and response to Fusarium solani infection. (A to C) The root rot disease index (A), stem length (B), and stem weight (C) were measured and subjected to analysis of variance (ANOVA) and Tukey’s test. Tukey’s test results were used to group samples based on statistically significant differences (*P < *0.05), as determined by using multcompView (version 1.4-20) in R. The vertical bars represent the standard errors of the means. (D and E) The F. solani density in the rhizosphere was analyzed by qPCR (D), and Pearson’s correlation was calculated between the F. solani density and the root rot disease index (E). (F) The F. solani dry weight was measured after 1 week of incubation in NFb liquid medium with different nitrogen sources. F. solani dry weights were analyzed using Welch’s ANOVA due to unequal variances, but normality was maintained. Error bars represent means ± standard errors. HSD, honestly significant difference.

The impact of repeated ginseng monoculture planting using the same soil was investigated previously (19), and it was observed that nitrogen-fixing microbial taxa were crucial in the early stages of the monoculture system. This study aimed to evaluate the effects of various nitrogen sources, including NH_4_Cl, early-stage nitrogen metabolism amino acids (Glu, Asp, and Asn), and a later-stage amino acid (Val), on the growth and root rot disease severity of ginseng. The results showed that the root rot disease severity index was highest for the Val treatment and lowest for the Asp treatment (Fig. 1A). With regard to ginseng length and weight, treatments with NH_4_Cl and Val were not favorable, while treatments with early-stage nitrogen metabolism amino acids led to relatively healthy states (Fig. 1B and C). The concentration of F. solani was found to be between 10^5^ and 10^7^ CFU/g of soil, with a positive correlation with the degree of disease progression (Fig. 1D and E), consistent with the findings from the 7th to 10th plantings in the previous study (19) (see Fig. S1 in the supplemental material). To further understand the impacts of different nitrogen sources on the growth of F. solani, dry weights were measured by mixing different nitrogen sources in nitrogen-free bromothymol blue malate broth (NFb) medium. The results showed that the level of growth of F. solani was highest with the Glu and Val treatments (Fig. 1F). These findings provide new insights into the role of nitrogen sources in the growth and root rot disease severity of ginseng and highlight the importance of early-stage nitrogen metabolism in maintaining healthy ginseng growth.

### Functional differences in the bacterial community.

**FIG 2:**
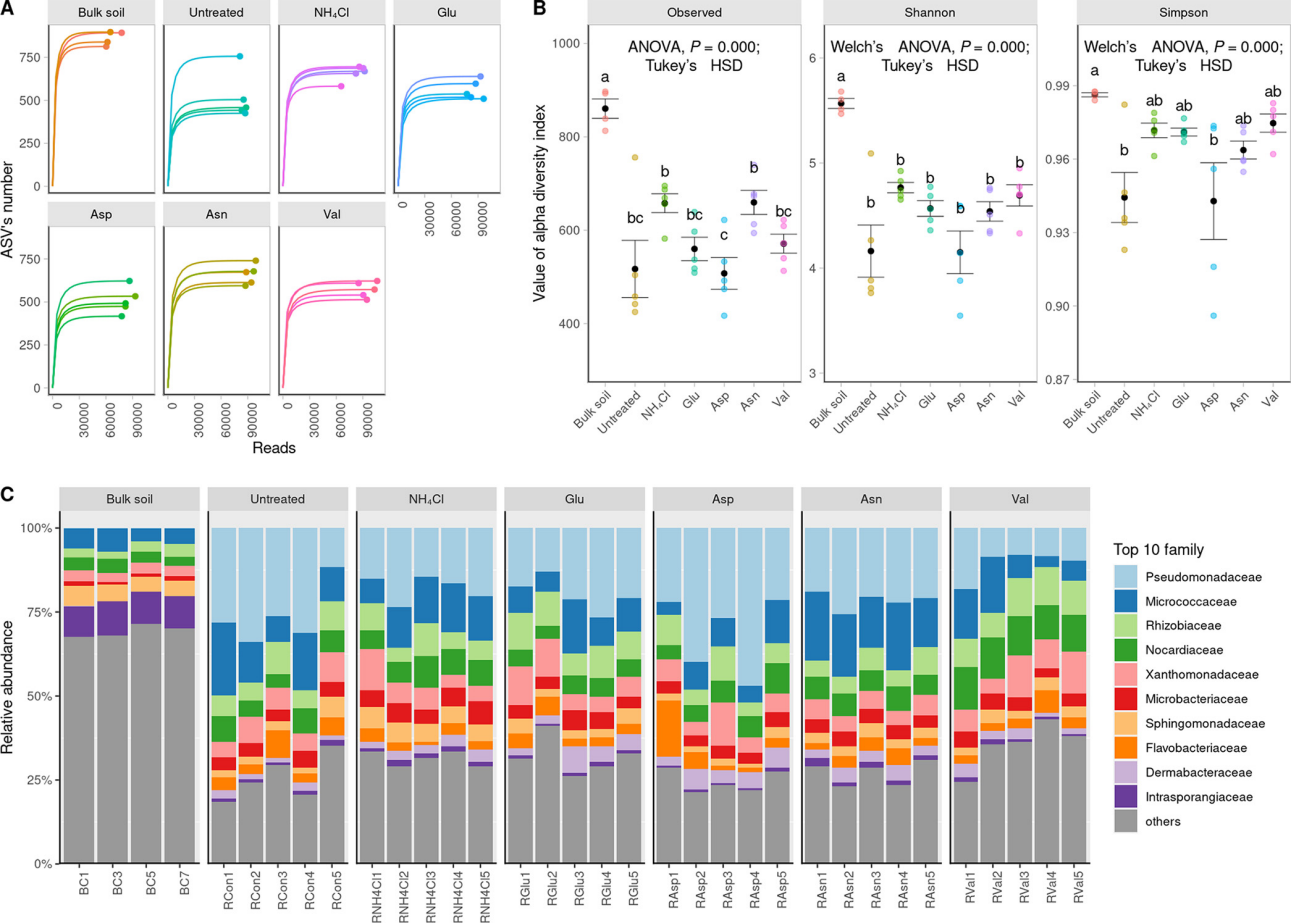
Diversity analysis of the 16S rRNA library. (A) Rarefaction curve showing that the interpolated read at the end of the curve is parallel to the *x* axis, indicating that sufficient reads have been obtained and that no additional amplicon sequence variants (ASVs) are expected to be observed. (B) α-Diversity was calculated using the Shannon and Simpson indices, which exhibited a normal distribution according to the Shapiro-Wilk test. The homogeneity or heterogeneity of the variance was determined using ANOVA or Welch’s ANOVA, respectively. *Post hoc* analysis was conducted using Tukey’s test, and multcompView was used to identify groups that were not significantly different (*P > *0.05). (C) Relative abundances of taxa at the family level. Only the top 10 most abundant families are displayed using different colors.

The population density of F. solani in soil alone cannot accurately explain the progression of root rot. To better understand this phenomenon, the microbiota community in the ginseng rhizosphere was observed. Amplicon sequence variant (ASV) clustering was paralleled to the end of the rarefaction curve on the *x* axis, and the α-diversity values and relative abundances at the family level were determined (Fig. 2A). The results indicated that α-diversity values were not significantly different among the treatments and differentiated between only the rhizosphere and bulk soil of untreated nitrogen (Fig. 2B). Overall, *Pseudomonadaceae* dominated the rhizosphere (Fig. 2C). Previous studies indicated that influencer bacteria such as *Beijerinckiaceae*, *Comamonadaceae*, *Devosiaceae*, *Rhizobiaceae*, *Sphingobacteriaceae*, *Sphingomonadaceae*, and *Xanthomonadaceae* play a significant role in bacterial community turnover (19). However, no significant differences in influencer taxa were observed among the different nitrogen sources (Fig. S2). Previous studies also noted that nitrogen fixation is a key function of influencer taxa; however, no differences in nitrogen fixation abilities were observed among different nitrogen sources, as determined by the gene owned by operational taxonomic units (OTUs) (GOO) index (calculated for *nifH*, *nifD*, and *nifK* for the production of the nitrogenase complex NifHDK). It should be noted that a direct comparison between the rhizosphere and bulk soil is difficult as the absolute abundances of bacteria will differ, but the nitrogen fixation ability was relatively high in the bulk soil (see Fig. S3 in the supplemental material).

**FIG 3:**
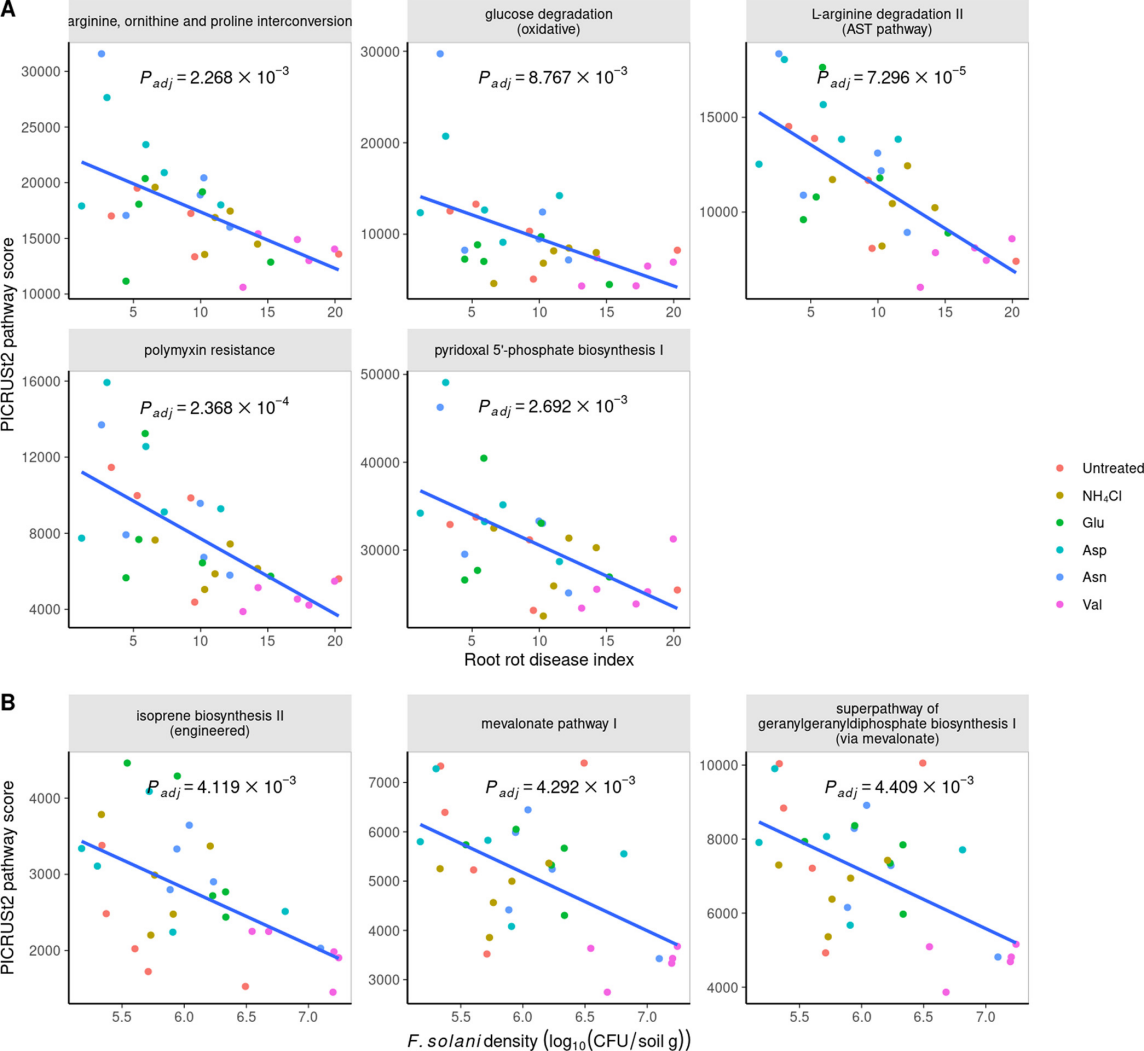
A negative correlation pathway was identified between the disease index and the F. solani density. Pearson correlation analysis was performed between 428 pathways and both the disease index and F. solani density. The significant pathways were determined using the FDR adjustment method with an adjusted *P* (*P*_adj_) value of <0.01.

Based on these findings, a new hypothesis was developed, suggesting that the density of F. solani may not be a critical factor in determining the severity of ginseng root rot. By analysis of Pearson’s correlation and false discovery rate (FDR) adjustment, it was determined that there were several microbiota metabolic pathways that were negatively correlated with the disease index and F. solani density. Out of the 428 pathways predicted by PICRUSt2 (Phylogenetic Investigation of Communities by Reconstruction of Unobserved States, version 2.3.0-b) using the MetaCyc database, 5 pathways that showed a negative correlation with the disease index and 3 pathways that showed a negative correlation with the F. solani density were selected (Fig. 3). The pathways selected were “arginine, ornithine, and proline interconversion”; “glucose degradation (oxidative)”; “l-arginine degradation II (arginine succinyltransferase pathway)”; “polymyxin resistance”; and “pyridoxal 5′-phosphate biosynthesis I” for their correlation with the disease index and “isoprene biosynthesis II (engineered),” “mevalonate pathway I,” and “superpathway of geranylgeranyldiphosphate biosynthesis (via mevalonate)” for their correlation with the F. solani density. The genes belonging to these pathways were then organized for comparison by phylogenetic group and by sample by a GOO index comparison (Table S2).

**FIG 4:**
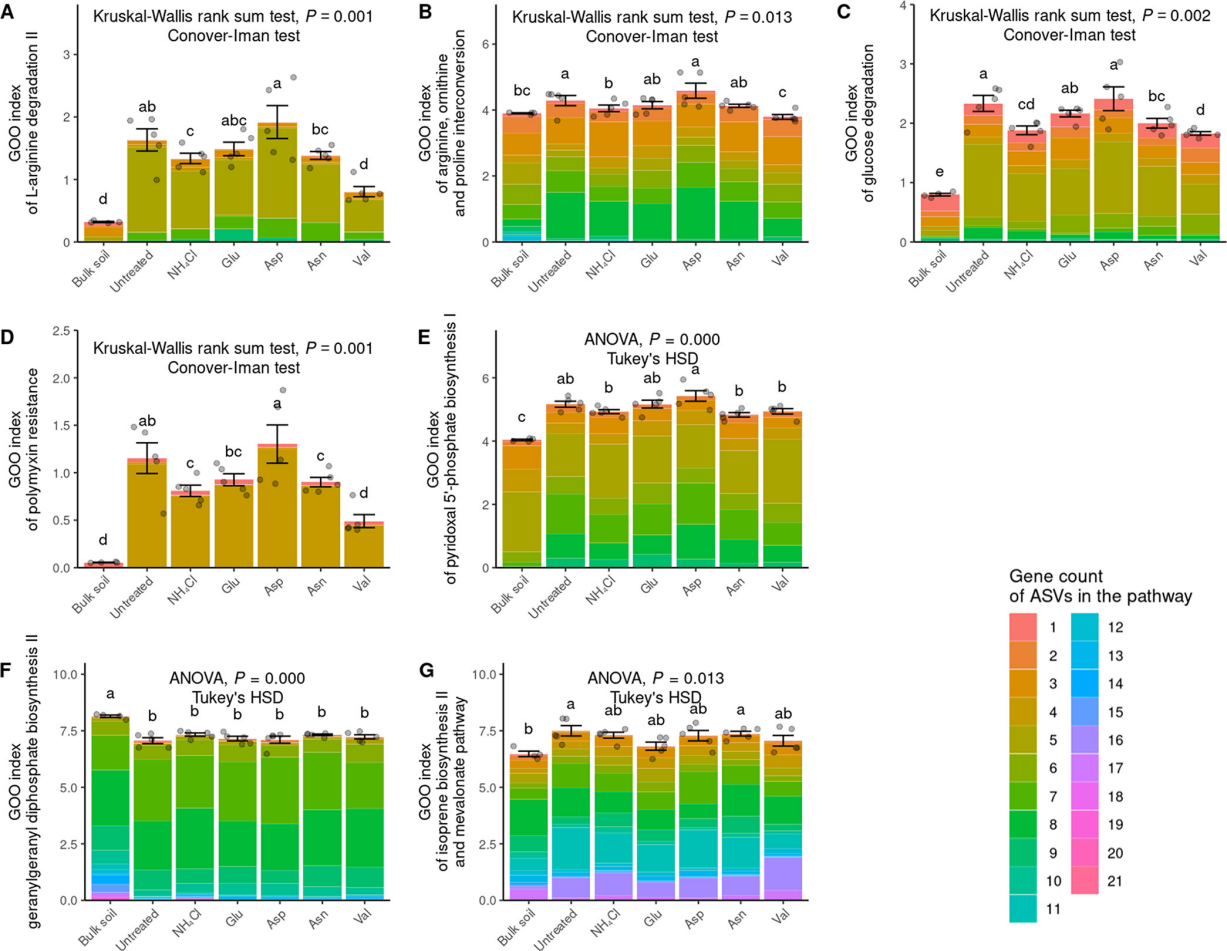
Correlation between the disease index and the density of F. solani illustrated by the GOO index. (A to F) The pathways with a negative correlation with the disease index, attributed to l-arginine degradation II (arginine succinyltransferase pathway) (A), and the involvement of other pathways, including arginine, ornithine, and proline interconversion (B); glucose degradation (oxidative) (C), polymyxin resistance (D), pyridoxal 5′-phosphate biosynthesis I (E), and the superpathway of geranylgeranyl diphosphate biosynthesis (F), are represented and were analyzed for their GOO indices. (G) Analysis of the pathway genes of the isoprene biosynthesis II (engineered) and mevalonate pathways, which overlap and were therefore analyzed together. Due to the commonality of many pathways, the sum of multiplications of the relative abundances and the numbers of contributing genes in the pathway was used for analysis. The GOO indices were compared by ANOVA, Welch’s ANOVA, or a Kruskal-Wallis rank sum test, depending on the normal distribution and homogeneity of variance based on the Shapiro-Wilk normality test and the Bartlett test. *Post hoc* analyses were conducted using Tukey’s honestly significant difference test and the Conover-Iman test. The results of the *post hoc* tests were grouped into no difference (*P > *0.05) using multcompView (version 1.4-20) in R.

The relative abundances of each ASV and its corresponding gene pathway were analyzed using the PICRUSt2 results. The gene counts belonging to each ASV and its corresponding pathway were compared with the relative abundance of each ASV (Fig. S4). The GOO index was calculated to determine the potential gene amount in a sample. The relative abundances of ASVs contributing to disease-negative pathways such as l-arginine degradation II; arginine, ornithine, and proline interconversion; glucose degradation; polymyxin resistance; and pyridoxal 5′-phosphate biosynthesis I were determined (Fig. 4). The ASV index is also described in Fig. 4. Some pathways were too common to be evaluated by relative abundance; therefore, the GOO index was used to evaluate them. For the Asp treatment with the least disease, the pathway-related functionality was highest except for glucose degradation. Conversely, in the Val treatment with the most disease, the lowest functionalities were observed for pathways such as l-arginine degradation II; arginine, ornithine, and proline interconversion; glucose degradation; and polymyxin resistance. The superpathway of geranylgeranyldiphosphate biosynthesis I (via mevalonate), the isoprene biosynthesis II (engineered) pathway, and mevalonate pathway I (eukaryotes and bacteria) were common characteristics of the pathways negatively correlated with the F. solani density. These pathways were part of terpenoid backbone synthesis. Isoprene biosynthesis II (engineered) and mevalonate pathway I were analyzed together due to their excessive gene overlap. These pathways were very common, and almost all bacteria contributed to them. No differences in the rhizosphere were observed when comparing the GOO indices for these pathways, regardless of the nitrogen source (Fig. 4).

### Pseudomonas, a keystone taxon in root rot suppression.

**FIG 5:**
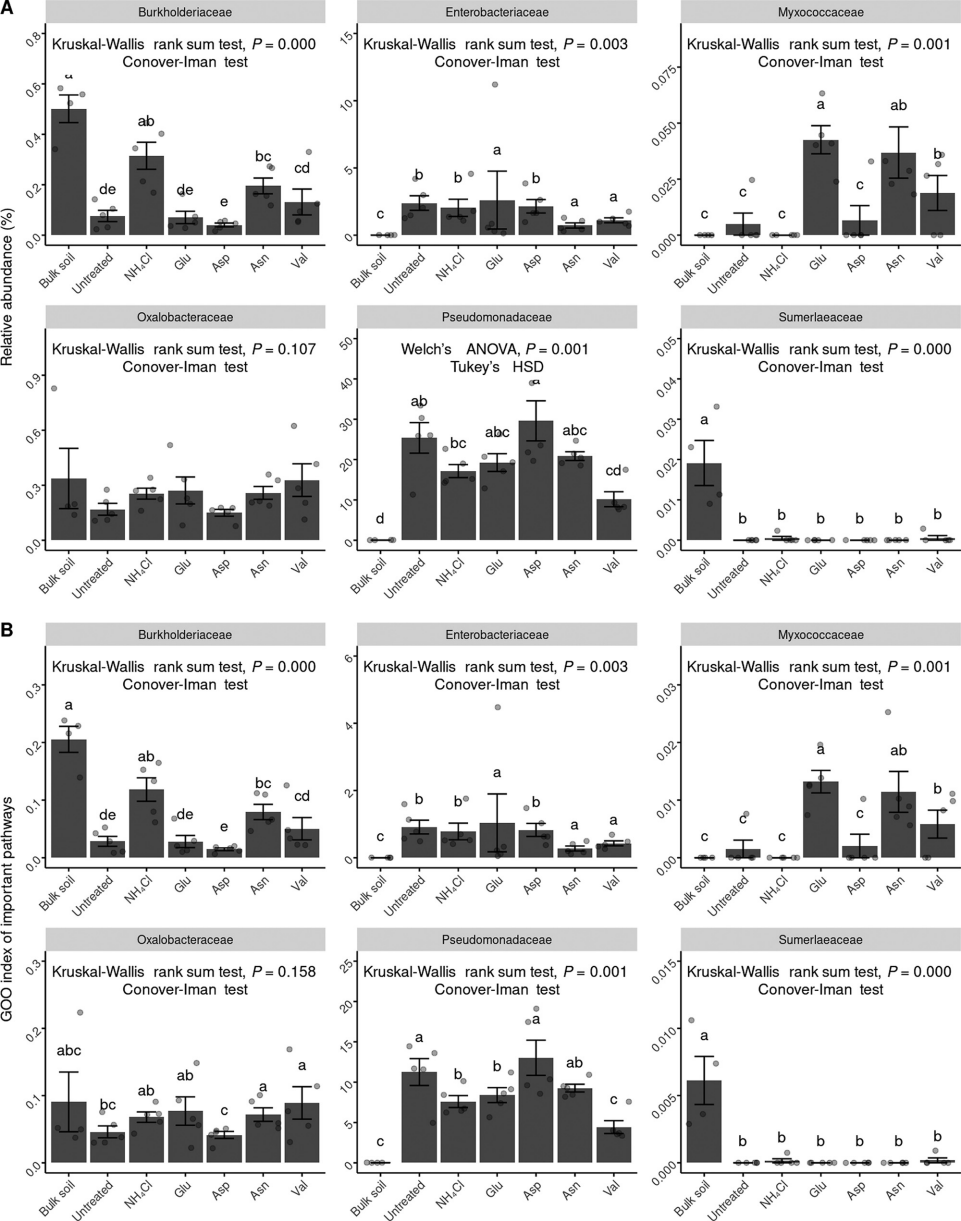
Relative abundances and GOO indices of taxa contributing to all eight pathways. The relative abundances (A) and the GOO indices (B) of ASVs with at least one gene in the eight pathways were determined. The taxonomic classification of the ASVs is limited to six families. *Pseudomonadaceae* exhibited the highest relative abundance and GOO index in the rhizosphere, and their properties were enhanced following Asp treatment but reduced following Val treatment. Statistical analysis was performed using the Kruskal-Wallis test with the Conover-Iman *post hoc* test.

**FIG 6:**
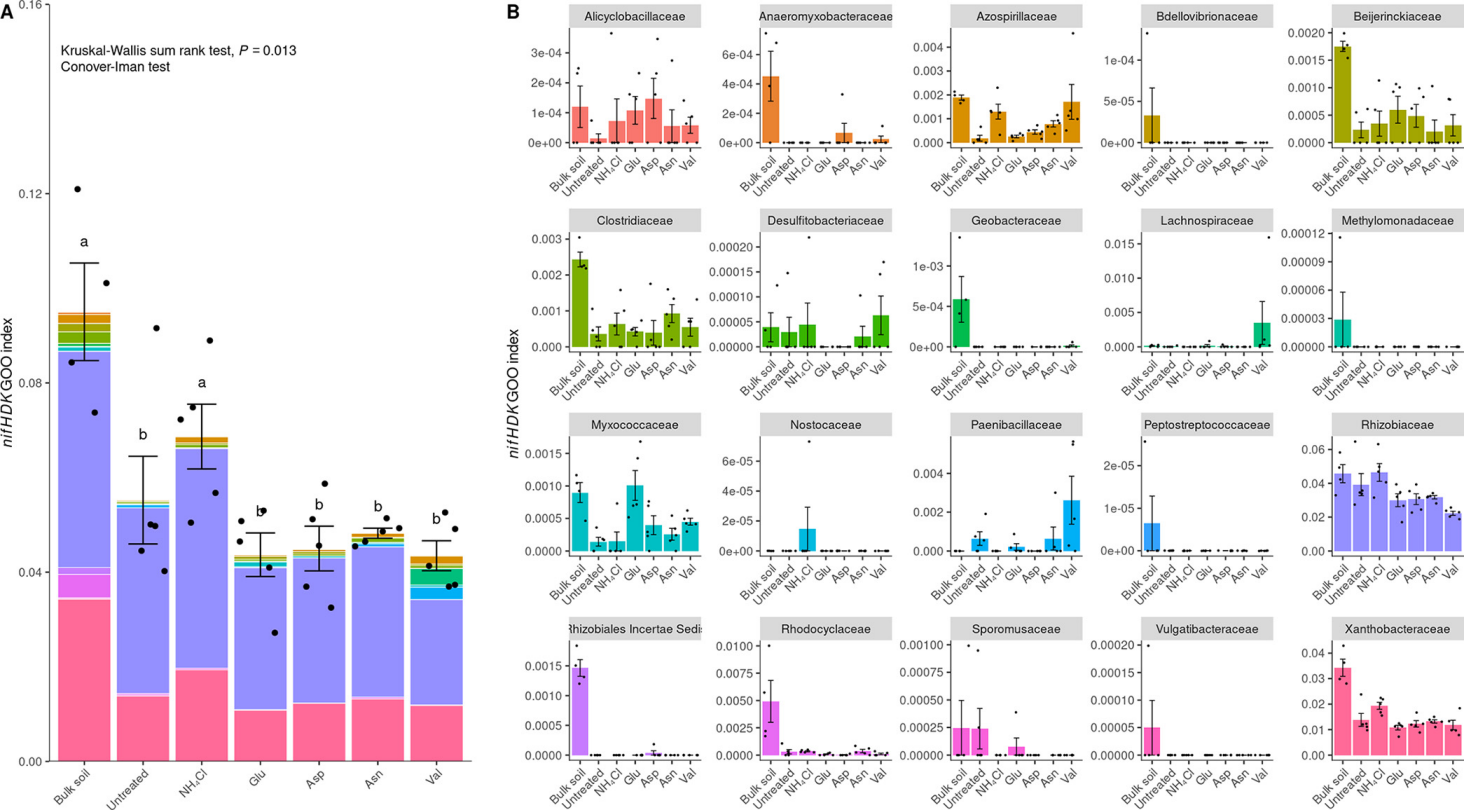
The GOO index was calculated for the *nifHDK* gene family in different soil samples. (A) Results at the sample level, with 20 taxa contributing to nitrogen fixation and the *nifHDK* GOO index being lower for amino acid treatments than in the bulk soil. Statistical analysis was conducted using the Kruskal-Wallis rank sum test and the Conover-Iman *post hoc* test. The *post hoc* test result is presented by the group based on undistinguished (*P* > 0). (B) Detailed information on the values for each taxon, as indicated in the key.

**FIG 7:**
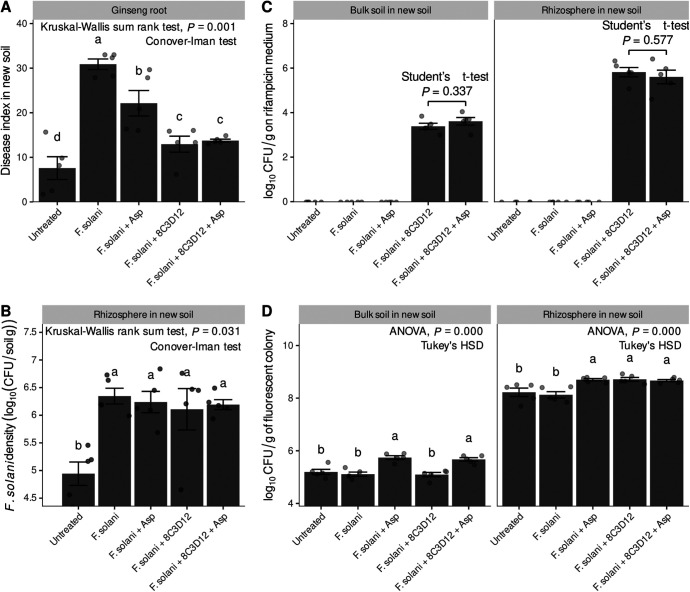
Effect of *Pseudomonadaceae* strain 8C3D12 on ginseng root rot occurrence and the F. solani population in the soil. Prior to the experiment, strain 8C3D12 was made rifampicin resistant (100 μg/mL). (A to C) The experiment included assessments of the root rot disease index (*n *= 150) (A); the *F. salami* density, quantified by qPCR (B); and the CFU of 8C3D12 in bulk soil and the rhizosphere, measured on King’s B medium containing 100 μg/mL rifampicin (C). The detection of the 8C3D12 colony was achieved by fluorescence using UV light (365 nm) in the dark. (D) Additionally, the total *Pseudomonadaceae* CFU were counted using fluorescence in bulk soil and the rhizosphere. Statistical analysis included comparisons of each measurement value using Student’s *t* test, ANOVA, and the Kruskal-Willis rank sum test, taking into account the number of groups, normal distribution, and equal variance. *Post hoc* analysis was performed using Tukey’s HSD test and the Conover-Iman test.

To determine the dominant microbial taxa contributing to the eight selected pathways of significance, a compilation of ASVs with at least one gene in each pathway was performed (Table S3). Upon examining the relative abundance of each ASV at the family level, it was found that the *Pseudomonadaceae* constituted 10 to 30% of the total, while all other families constituted less than 3% or less than 0.1%. The relative abundance of the *Pseudomonadaceae* was highest for the Asp treatment and lowest for the Val treatment (Fig. 5A). In terms of the GOO indices for the eight pathways, the *Pseudomonadaceae* displayed the highest range, from 5 to 12, while all other families showed low values, less than 1. The highest GOO index for the *Pseudomonadaceae* was observed for the Asp treatment, and the lowest GOO index was observed for the Val treatment (Fig. 5B). Considering that the ASVs of the *Pseudomonadaceae* were largely comprised of the genus Pseudomonas (Fig. S5), a Pseudomonas strain (8C3D12), which was obtained from the rhizosphere, was found to closely match the V4 sequence of ASV Sq_1, the most dominant ASV in the rhizosphere (Table 1 and Fig. 6). In a root rot disease suppression assay in pots, strain 8C3D12 showed a greater ability to prevent root rot disease than the Asp treatment. However, no significant differences were observed between the combination 8C3D12-Asp treatment and 8C3D12 treatment alone (Fig. 7A). No changes in the density of F. solani in the pot were observed (Fig. 7B). Additionally, both 8C3D12 and other Pseudomonas strains demonstrated reliable root colonization abilities, and the Asp treatment was found to promote the dominance of Pseudomonas in the bulk soil (Fig. 7C and D).

**TABLE 1:** Results of BLASTn comparisons for the top 10 resemblances between the 8C3D12 strain 16S rRNA and ASVs

ASV ID	Identification by IDTAXA	Mean relative abundance in rhizosphere (%) ± SD	BLASTn result with the 8C3D12 strain			
			Score	E value	No. of colonies with identity to 8C3D12/total no. of colonies (%)	No. of colonies with gaps/total no. of colonies (%)
Sq_1	Genus Pseudomonas	11.07 ± 5.78	468	7e−133	253/253 (100)	0/253 (0)
Sq_694	Family *Pseudomonadaceae*	0.01 ± 0.05	462	3e−131	252/253 (99)	0/253 (0)
Sq_86	Genus Pseudomonas	0.21 ± 0.38	462	3e−131	252/253 (99)	0/253 (0)
Sq_23	Genus Pseudomonas	0.71 ± 0.02	451	7e−128	251/254 (99)	2/254 (1)
Sq_445	Genus Pseudomonas	0.02 ± 0.06	446	3e−126	250/254 (98)	2/254 (1)
Sq_14	Genus Pseudomonas	1.44 ± 1.42	446	3e−126	249/253 (98)	0/253 (0)
Sq_3	Genus Pseudomonas	5.19 ± 5.25	446	3e−126	250/254 (98)	2/254 (1)
Sq_28	Genus Pseudomonas	0.55 ± 1.60	440	2e−124	249/254 (98)	2/254 (1)
Sq_18	Genus Pseudomonas	0.82 ± 0.78	440	2e−124	249/254 (98)	2/254 (1)
Sq_160	Genus Pseudomonas	0.09 ± 0.21	440	2e−124	248/253 (98)	0/253 (0)

## DISCUSSION

In a previous study of a ginseng monoculture model system, it was observed that the initial bacterial community structure in the rhizosphere collapsed at a conducive stage and that nitrogen fixation was an important ability for influential taxa to maintain the microbiota community structure (19). The present study aimed to investigate how treatment with nitrogen sources affects ginseng communities that have deteriorated due to the continuous cultivation of ginseng. For nitrogen treatment, the severity of root rot disease was lowest for the Asp treatment, while it was the most severe for the Val treatment. The occurrence of severe disease with the Val treatment may be attributed to the enhancement of the growth of F. solani in the soil. However, the reason for the relief of disease with the Asp treatment remains inadequately explained as F. solani grew well in NFb with Asp. The current study showed a positive correlation between the disease index and the population density of F. solani, which remained consistent even in the 7th to 10th plantings, which was the last condition of serial cultivation in the previous study (see Fig. S3 in the supplemental material). This study demonstrated that the experimental design was heavily influenced by the microbiota community after the outbreak of root rot disease.

To investigate the interaction between microbiota communities and root rot disease, a community analysis based on a 16S rRNA library was conducted. Surprisingly, the initial community influencers, including *Beijerinckiaceae*, *Comamonadaceae*, *Devosiaceae*, *Rhizobiaceae*, *Sphingobacteriaceae*, *Sphingomonadaceae*, and *Xanthomonadaceae*, which dominated and promoted nitrogen fixation in the early stages of continuous ginseng cultivation and interfered with the root rot outbreak, did not differ in the rhizosphere when treated with the amino acids. To evaluate the nitrogen fixation abilities of these influencer taxa, the GOO index for *nifH*, *nifD*, and *nifK*, which encode the nitrogenase complex NifHDK, was calculated. The GOO index was not significantly different in the rhizosphere. Among the initial influencer taxa, only *Rhizobiaceae* and *Beijerinckiaceae* possessed the *nifHDK* genes, while the other influencers did not. The absence of *nifHDK* detection in the other influencer taxa may be a limitation of the PICRUSt2 database. Nevertheless, despite previous studies, these results suggest that the ability for nitrogen fixation or the abundance of initial influencers, which was independent of the disease index, indicates the lesser importance of nitrogen fixation in the initial stage of continuous cultivation.

In order to elucidate the origin of the disease control ability of the initial community influencer taxa, we examined negative correlations between the community metabolic pathways and both the root rot disease index and the density of F. solani. Among the pathways with a negative correlation with the disease index, the l-arginine degradation II and arginine, ornithine, and proline interconversion pathways have a common feature in that they generate ammonium and l-glutamate from arginine. Ammonium produced by nitrogen fixation reacts with glutamate during the conversion of amino acids to glutamine (21). Therefore, the two pathways possess opposing properties of producing ammonium from amino acids. The final product of the pyridoxal 5′-phosphate biosynthesis I pathway, pyridoxal 5′-phosphate (PLP), is the active form of vitamin B_6_ (pyridoxine), which serves as a cofactor for essential enzymes involved in the metabolism of macronutrients such as carbohydrates, amino acids, and lipids (22). The glucose degradation (oxidative) pathway converts d-glucopyranose, a ring form of glucose, into d-gluconate 6-phosphate before producing pyruvate, which is required for the tricarboxylic acid (TCA) cycle (23). The polymyxin resistance pathway produces a lipopolysaccharide (LPS) called 4′-α-l-Ara4N-α-Kdo-(2→4)-α-Kdo-(2→6)-[P4′-α-l-ara4N]-lipid A from 4-amino-4-deoxy-l-arabinopyranose (24). It has been reported that positively charged polymyxin is less likely to interact with cell membranes that are highly negatively charged due to the presence of LPS. Among the pathways, the l-arginine degradation II; arginine, ornithine, and proline; and glucose degradation (oxidative) pathways were presumed to be primary metabolism pathways that could help with direct or indirect root rot suppression or be characteristic of root rot suppression bacteria. The polymyxin resistance pathway may confer a competitive advantage within the microbiota community. In the pyridoxal 5′-phosphate biosynthesis I pathway, PdxH, which converts pyridoxin 5′-phosphate to PLP, is homologous to PhzG, an enzyme involved in the biosynthesis of the antifungal compound phenazine carboxylic acid (25). The GOO indices for these five disease-negative pathways were higher in untreated soil than in bulk soil, indicating that ginseng actively promoted the growth of bacteria with these traits. Furthermore, the GOO index was highest for the Asp treatment and lowest for the Val treatment except for the glucose degradation (oxidative) pathway. This GOO pattern and disease index (DI) pattern were found to be perfectly inverse, suggesting that bacteria in the rhizosphere can selectively grow using specific nitrogen sources, which affects disease progression.

The pathways that were negatively correlated with the density of F. solani shared a feature of terpenoid backbone pathways. These pathways included the nearly overlapping mevalonate pathway I and isoprene biosynthesis II (engineered) pathway, which produced the shortest terpenoid backbone, isopentenyl pyrophosphate (IPP) or dimethylallyl pyrophosphate (DMAPP). The final molecule, geranylgeranyldiphosphate, in the geranylgeranyldiphosphate biosynthesis (via mevalonate) pathway was known to be formed by combining one DMAPP and three IPP molecules (26). While there was no difference in the GOO indices among different rhizospheres, it was observed that ginseng multiplied bacteria that produced longer terpenoid backbones than shorter ones in both untreated and bulk soil. This maintained a more diverse concentration of secondary metabolites around the ginseng. Therefore, although the GOO index was not affected by different nitrogen sources in the rhizosphere, we should not overlook the possibility of various secondary metabolites with antifungal activity from terpenoids, which were unpredictable by PICRUSt2.

To gain insight into the primary contributors to the eight essential pathways, we identified ASVs containing at least one gene associated with each pathway. The ASVs were classified into six phylogenetic groups: *Burkholderiaceae*, *Enterobacteriaceae*, *Myxococcaceae*, *Oxalobacteraceae*, *Pseudomonadaceae*, and “*Candidatus* Sumerlaeaceae.” Within these groups, *Pseudomonadaceae* exhibited a significantly higher relative abundance and GOO index for all eight pathways. Notably, *Pseudomonadaceae* had the highest values for both the Asp and Val treatments, which corresponded to the pattern of inverted root rot disease. Based on these findings, we determined that the family *Pseudomonadaceae* is the most important taxon for suppressing ginseng root rot disease.

In order to demonstrate the importance of *Pseudomonadaceae*, a strain known as 8C3D12 was isolated from the endosphere during the 8th cycling stage of the ginseng monoculture model system. The 16S rRNA V4 sequence of ASV Sq_1 was found to perfectly match that of 8C3D12, and Sq_1 was the most abundant ASV in the rhizosphere. After conferring rifampicin resistance to 8C3D12, the bacterium was introduced to new soil and used to inoculate ginseng plants. 8C3D12 was found to effectively colonize the rhizosphere, resulting in healthier ginseng than those treated with F. solani and Asp. However, the combination of 8C3D12 and Asp treatments did not exhibit a noticeable difference compared to the single treatment with 8C3D12. It was determined that the Asp treatment contributed to the overall dominance of Pseudomonas in the bulk soil. While 8C3D12 treatment was sufficient to establish dominance in the rhizosphere, Asp treatment did not assist in root colonization by 8C3D12. Additionally, Asp and 8C3D12 treatments did not affect the growth of F. solani. These results were consistent with the Pearson correlation outcome of the GOO index, which exhibited a negatively correlated pathway. Tests on new soils, which were not involved in the bacterial communities, clearly indicated that Asp treatment inhibited root rot and was associated with an increase in Pseudomonas. These findings suggest that 8C3D12 significantly aids in the inhibition of root rot disease. Furthermore, a correlation was observed between the density of F. solani and the disease index in the nitrogen treatment test and the 7th to 10th plantings in the previous study (19). However, it was evident that Pseudomonas did not inhibit growth and that other factors inhibited the growth of F. solani, which was not recognized in this study. In the case of 8C3D12, which shows a perfect match with Sq_1, it was interesting that it was almost nonexistent in bulk soil but was highly abundant in the rhizosphere. Considering that it was isolated from the endosphere, one hypothesis could be that this Pseudomonas strain was very dependent on the host and may interact directly with plants to modulate plant immunity.

**FIG 8:**
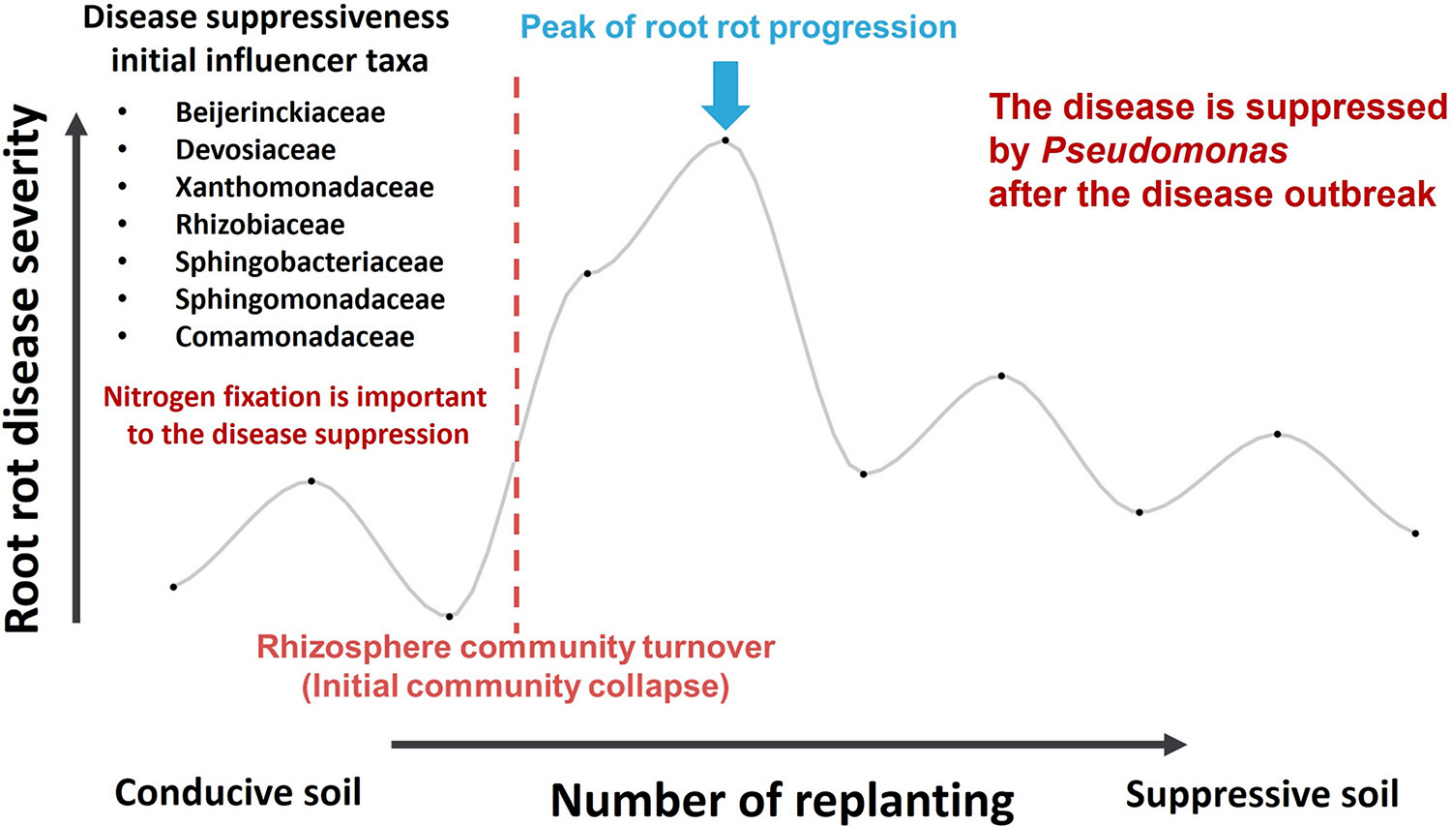
Suppression of root rot disease by Pseudomonas occurs following the peak of root rot disease progression.

These findings elucidated that the incorporation of Asp as the primary nitrogenous fertilizer component by Pseudomonas could potentially aid in managing root rot disease in ginseng cultivation. It has been suggested that while the application of nitrogen-rich fertilizers may be crucial during disease outbreaks, the driving force behind disease control may shift from the initial microbiota community to specific influencers such as Pseudomonas during continuous field cultivation. Following disease outbreaks in continuous monocultures, suppressive soil may develop (14), and several cases have been reported wherein Pseudomonas spp. have been implicated in the formation of suppressive soil (15). The current investigation highlights the significance of Pseudomonas in the context of suppressive soil formation after disease outbreaks, thereby aiding in a better understanding of this phenomenon. Moreover, the findings of this study demonstrate the progressive changes in specific suppression induced by Pseudomonas, beginning from the collapse of the initial microbiota community to the disappearance of general suppression and a disease outbreak (Fig. 8).

## MATERIALS AND METHODS

### Nitrogen source treatment and phenotypic characteristics of ginseng.

A soil sample was collected from a 9-year-old ginseng monoculture field (GPS coordinates, 36°56′34.9″N, 127°44′59.7″E). The raw soil (10%) was mixed with 50% autoclaved sand and 40% autoclaved raw soil to create a growth medium for the ginseng seedlings. This mixture was used for 10 cycles of ginseng monoculture cultivation. After 10 cycles, the ginseng plants were grown on a mixture of 90% autoclaved sand and 10% continuously used soil, which showed a transformed bacterial community compared to the initial planting phase in the monoculture system (19) (see Fig. S6 in the supplemental material). The mixed soil was placed on top of pebbles in pots, and 25 ginseng seedlings without leaves were planted and cultured for 20 days. On the 0th and 10th days, the pots were treated with different nitrogen sources: 30 mL of distilled water, 50 mM NH_4_Cl, 50 mM Glu, 50 mM Asp, 50 mM Asn, and 50 mM Val (Fig. S6). After 20 days, the stem length, shoot length, and sprout length were measured, and each ginseng plant was scored for root rot disease severity. The severity of root rot disease was divided into five categories: no root rot, fine root rot, <10% rot, 10 to 50% rot, and 50 to 100% rot. Root rot severity was scored as 0, 1, 35, or 75, and the average score was used as the disease index at the pot level (Fig. S7). All experiments were done with three biological replicates (*n *= 25).

### Fusarium growth in different nitrogen sources.

To evaluate the effects of different nitrogen sources on the growth of Fusarium solani, the pathogen was cultured in glass tubes containing 5 mL of nitrogen-free bromothymol blue malate broth (NFb). NFb consisted of 5 g malic acid, 0.5 g K_2_HPO_4_, 0.5 g MgSO_4_·7H_2_O, 0.1 g NaCl, 0.02 g CaCl_2_·2H_2_O, 4.5 g KOH, 0.080 mg CuSO_4_·5H_2_O, 0.024 mg ZnSO_4_·7H_2_O, 2.8 mg H_3_BO_3_, 2 mg NaMoO_4_·2H_2_O, 2.35 mg MnSO_4_·H_2_O, 10 mg bromothymol blue, 65.6 mg Fe(III)-EDTA, 0.1 mg biotin, and 0.2 mg pyridoxal-HCl, dissolved in 1 L of distilled water. The pH of NFb was adjusted to 6.5 using KOH and HCl. The tubes were incubated in a shaking incubator at 28°C with a shaking speed of 170 rpm for 7 days. In each treatment group, NFb was supplemented with 50 mM NH_4_Cl, Glu, Asp, Asn, and Val, except for the untreated group. After incubation, the mycelium of F. solani was harvested by centrifugation and dried at 60°C for 2 days. The dried F. solani was then weighed to determine its growth.

### Rhizosphere collection and DNA extraction.

The rhizosphere was collected from 25 ginseng roots in a pot after shaking off the bulk soil. Through the detailed process described below, 5 rhizospheres were obtained using 5 pots for each nitrogen treatment. The roots were sonicated in a sterile glass beaker containing 200 mL of prechilled phosphate-buffered saline (PBS) in a sonicating bath for 20 min. The PBS solution was comprised of 8 g NaCl, 200 mg KCl, 1.44 g Na_2_HPO_4_, and 245 mg KH_2_PO_4_ dissolved in 1 L of water, with a pH of 7.4. The rhizosphere was settled by centrifugation using a 1736R centrifuge (LaboGene, Seoul, Republic of Korea) at 3,000 × *g* for 20 min. After the removal of the supernatant, the rhizosphere was stored at −80°C for further analysis. The extraction of DNA from the rhizosphere was performed using the FastDNA Spin kit for soil (MP Biomedicals, Solon, OH, USA). Approximately 500 mg of the rhizosphere was used for the extraction process. The rhizosphere was mixed with 978 μL of sodium phosphate buffer and 122 μL of MT buffer and homogenized using the FastPrep-24 classic instrument (MP Biomedicals, Solon, OH, USA) at a speed of 6.0 for 40 s. The resulting supernatant was obtained by centrifugation at 18,000 × *g* for 10 min, transferred to a clean 2-mL tube, and combined with 250 μL of protein precipitation solution. The mixture was inverted 10 times and centrifuged again to obtain the supernatant. The supernatant was then transferred to a 15-mL conical tube, combined with 1 mL of the resuspended binding matrix, and inverted for 2 min before settling for 3 min. The top 500 μL of the settled mixture was discarded, and the remaining matrix was collected by suspension and centrifugation using the Spin system. The matrix was then washed with ethanol, and DNA was extracted by the addition of 50 μL of DNase-free water. As a control, bulk soil DNA was obtained prior to ginseng planting using the same method.

### Quantitative PCR for F. solani.

To quantify the population density of F. solani in the rhizosphere (Fig. S8), a quantitative PCR (qPCR) assay was performed using the SYBR green real-time PCR master mix (Toyobo, Osaka, Japan) and CFX Connect (Bio-Rad, CA, USA). The primer set used was ITS1F (5′-CTTGGTCATTTAGAGGAAGTAA-3′) as the forward primer and AFP346 (5′-GGTATGTTCACAGGGTTGATG-3′) as the reverse primer, as described previously by Lievens et al. (27). Standard DNA was extracted from an F. solani sand stock at a concentration of 10^7^ CFU/mL. The qPCR conditions were as follows: an initial denaturation step for 1 min at 95°C followed by 45 cycles of 15 s at 95°C, 15 s at 60°C, and 30 s at 72°C. Fluorescence was measured at the end of each cycle and during the melting-curve analysis, which was performed by reheating the reaction mixture to 95°C and cooling it to 60°C (Fig. S9).

### 16S rRNA V4 library sequencing.

Rhizosphere and bulk soil DNA samples were amplified using Kapa HiFi HotStart ReadyMix (Kapa Biosystems, Wilmington, MA, USA) and the Illumina adapter-attached primers 515F (5′-TCGTCGGCAGCGTCAGATGTGTATAAGAGACAGGTGCCAGCMGCCGCGGTAA-3′) and 805R (5′-GTCTCGTGGGCTCGGAGATGTGTATAAGAGACAGGACTACHVGGGTATCTAATCC-3′). The PCR amplification conditions were as follows: 95°C for 3 min followed by 25 cycles of 95°C for 30 s, 55°C for 30 s, and 72°C for 30 s, with a final extension step at 72°C for 5 min. The subsequent sequencing procedures were performed by Macrogen (Seoul, Republic of Korea). Linker and barcode sequences were added to the library using a thermal PCR protocol of 95°C for 3 min followed by 8 cycles of 95°C for 30 s, 55°C for 30 s, and 72°C for 30 s, with a final extension step at 72°C for 5 min. The library was then sequenced on a MiSeq 2× 300-bp platform (Illumina, San Diego, CA, USA).

### *In silico* analyses of the microbiota.

The *in silico* analyses were conducted on an Ubuntu 18.04-based workstation assembled with an AMD Ryzen Threadripper 3970X processor and 64-GB DDR4 RAM. Raw sequencing data in the FASTQ format were processed using the DADA2 (version 1.16.0) package in R (version 4.0.3). Reads with an average quality score of 30 or lower at the 3′ end were trimmed, and the error rate of the reads was estimated using the divisive amplicon denoising algorithm (DADA) (28). Subsequently, the forward and reverse reads were merged and clustered into amplicon sequence variants (ASVs) using the DADA2 algorithm. The chimeric ASVs were then removed. Taxonomic classification was performed using the IDTAXA algorithm (29), which is referred to in the SILVA 138 SSU (small-subunit) database. The resulting ASVs were classified using a naive Bayesian classifier in DECIPHER (version 2.16.1). The number of reads after each step of the process is provided in Table S1.

Microbiota community construction was analyzed using R packages such as vegan (version 2.5-0 [https://github.com/vegandevs/vegan]) and phyloseq (version 1.32.0 [https://github.com/joey711/phyloseq]), among others. To determine the relative abundances of genes in the samples or taxonomic groups, information obtained from 16S rRNA library sequences was converted to whole-metagenome shotgun sequencing results using PICRUSt2 (Phylogenetic Investigation of Communities by Reconstruction of Unobserved States, version 2.3.0-b) (30) in Python (version 3.7.8). The gene owned by operational taxonomic units (OTUs) (GOO) index was used to compare the functional group involvements of OTUs based on the presence of genes. The relative abundance of an OTU in a sample is represented by *A_o_*, while the number of functional group genes owned by an OTU is represented by *G_o_*: GOO = ∑*A_o_* × *G_o_*. In this study, the involvement of bacterial groups in gene groups was quantified by freely altering the OTUs and gene grouping units used for calculating the sum. The OTUs were clustered using the ASV method with DADA2, and the relative abundances and gene numbers of the ASVs were used. The contribution of a gene group to a metabolic pathway in a sample was evaluated using the GOO index calculated from the PICRUSt2 results of the 16S rRNA library analysis. The processed visual information was based on ggplot2 version 3.3.2 software (https://github.com/tidyverse/ggplot2). The R code used in this study is available in the GitHub repository (https://github.com/gyeongjunCho/R-code-of-Ph.D.-Thesis).

### Securing a keystone Pseudomonas strain and root rot disease suppression.

The family *Pseudomonadaceae* in the DADA2 ASV clustering results was represented mainly by Pseudomonas spp. A fluorescent colony was collected from the endophyte collection stock in the previous study (19), and the rhizosphere sample was plated onto peptone gelatin sucrose agar medium (10 g peptone, 20 g sucrose, 1 g K_2_HPO_4_, 1 g MgSO_4_·7H_2_O, 20 g gelatin, and 15  g agar per L) containing 0.002% (wt/vol) conalbumin (31). Out of approximately 300 colonies, 10 isolates were selected, and their 16S rRNA genes were sequenced. Only one strain was found to perfectly match the V4 region of Sq_1, which was commonly the most abundant genus in the rhizosphere (Table 1). This strain was derived from the endosphere and was named 8C3D12. The ability of 8C3D12 to prevent rot root disease in ginseng was tested in new soil with 1-year-old ginseng plants. Macroconidia (1 × 10^5^ conidia/g of soil) and microconidia (1 × 10^6^ conidia/g of soil) of F. solani were treated in a pot planted with 30 ginseng plants. The treatment density of 8C3D12, which was made resistant to rifampicin (100 μg/mL), was 4 × 10^5^ CFU/g of soil. Thirty milliliters of Asp (50 mM) was added on the 0th and 10th days. On the 20th day, the root rot disease index, F. solani density by qPCR, 8C3D12 density using King’s B agar medium with rifampicin (100 μg/mL), and Pseudomonas density using King’s B agar medium began to be measured. Pseudomonas and 8C3D12 for the density measurement were incubated at 27°C for 3 days and observed in the dark under UV light (365 nm), utilizing the fluorescence characteristics of the organisms.

### Data availability.

All data have been incorporated into the article and its supplemental material. The sequencing data used in this study were deposited in the BioProject database under accession number PRJNA971514.

## References

[1] Hong JT, Lee M-J, Yoon SJ, Shin SP, Bang CS, Baik GH, Kim DJ, Youn GS, Shin MJ, Ham YL, Suk KT, Kim B-S. 2021. Effect of Korea red ginseng on nonalcoholic fatty liver disease: an association of gut microbiota with liver function. J Ginseng Res 45:316–324. doi:10.1016/j.jgr.2020.07.004. 33841012 PMC8020261

[2] Xu T, Wan Y, Zhang J, Liu Q. 2018. Why ginseng has protective functions on the heart. Eur J Prev Cardiol 25:1150–1151. doi:10.1177/2047487318768943. 29629835

[3] Lee D-Y, Park CW, Lee SJ, Park H-R, Kim SH, Son S-U, Park J, Shin K-S. 2019. Anti-cancer effects of *Panax ginseng* berry polysaccharides via activation of immune-related cells. Front Pharmacol 10:1411. doi:10.3389/fphar.2019.01411. 32038228 PMC6988799

[4] Saba E, Jeong D, Irfan M, Lee YY, Park S-J, Park C-K, Rhee MH. 2018. Anti-inflammatory activity of rg3-enriched Korean red ginseng extract in murine model of sepsis. Evid Based Complement Alternat Med 2018:6874692. doi:10.1155/2018/6874692. 30405742 PMC6201491

[5] Ichim MC, de Boer HJ. 2021. A review of authenticity and authentication of commercial ginseng herbal medicines and food supplements. Front Pharmacol 11:612071. doi:10.3389/fphar.2020.612071. 33505315 PMC7832030

[6] Kim Y-S, Lee M-S, Yeom J-H, Song J-G, Lee I-K, Yeo W-H, Yun B-S. 2012. Screening of antagonistic bacteria for biological control of ginseng root rot. Kor J Mycol 40:44–48. doi:10.4489/KJM.2012.40.1.044.

[7] Park K. 2001. Fitness analysis of the forecasting model for root rot progress of ginseng based on bioassay and soil environmental factors. Res Plant Dis 7:20–24.

[8] Punja ZK, Wan A, Goswami RS, Verma N, Rahman M, Barasubiye T, Seifert KA, Lévesque CA. 2007. Diversity of *Fusarium* species associated with discolored ginseng roots in British Columbia. Can J Plant Pathol 29:340–353. doi:10.1080/07060660709507480.

[9] Punja ZK, Wan A, Goswami RS. 2008. Root rot and distortion of ginseng seedling roots caused by *Fusarium oxysporum*. Can J Plant Pathol 30:565–574. doi:10.1080/07060660809507556.

[10] Lee S-G. 2004. *Fusarium* species associated with ginseng (*Panax ginseng*) and their role in the root-rot of ginseng plant. Res Plant Dis 10:248–259. doi:10.5423/RPD.2004.10.4.248.

[11] Cook RJ, Rovira A. 1976. The role of bacteria in the biological control of *Gaeumannomyces graminis* by suppressive soils. Soil Biol Biochem 8:269–273. doi:10.1016/0038-0717(76)90056-0.

[12] Weller DM, Raaijmakers JM, Gardener BBM, Thomashow LS. 2002. Microbial populations responsible for specific soil suppressiveness to plant pathogens. Annu Rev Phytopathol 40:309–348. doi:10.1146/annurev.phyto.40.030402.110010. 12147763

[13] Schlatter D, Kinkel L, Thomashow LS, Weller D, Paulitz T. 2017. Disease suppressive soils: new insights from the soil microbiome. Phytopathology 107:1284–1297. doi:10.1094/PHYTO-03-17-0111-RVW. 28650266

[14] Kwak YS, Weller DM. 2013. Take-all of wheat and natural disease suppressive: a review. Plant Pathol J 29:125–135. doi:10.5423/PPJ.SI.07.2012.0112. 25288939 PMC4174779

[15] Yang M-M, Mavrodi DV, Mavrodi OV, Bonsall RF, Parejko JA, Paulitz TC, Thomashow LS, Yang H-T, Weller DM, Guo J-H. 2011. Biological control of take-all by fluorescent *Pseudomonas* spp. from Chinese wheat fields. Phytopathology 101:1481–1491. doi:10.1094/PHYTO-04-11-0096. 22070279

[16] Hiltner L. 1904. Über neure Erfahrungen und probleme auf dem gebeit der bodenbackteriologie und unter besonderer berucksichtigung der grundungung und brache. Arb Dtsch Landwirtsch Ges 98:59–78.

[17] Čatská V. 2001. Pinton, R., Varanini, Z., Nannipieri, P. (ed.): The rhizosphere. Biochemistry and organic substances at the soil-plant interface. Biol Plant 44:372. doi:10.1023/A:1012468910553.

[18] Singh BK, Millard P, Whiteley AS, Murrell JC. 2004. Unravelling rhizosphere-microbial interactions: opportunities and limitations. Trends Microbiol 12:386–393. doi:10.1016/j.tim.2004.06.008. 15276615

[19] Cho G. 2021. PhD dissertation, Gyeongsang National University, Jinju, Republic of Korea.

[20] Lancien M, Gadal P, Hodges M. 2000. Enzyme redundancy and the importance of 2-oxoglutarate in higher plant ammonium assimilation. Plant Physiol 123:817–824. doi:10.1104/pp.123.3.817. 10889231 PMC1539263

[21] Miflin BJ, Habash DZ. 2002. The role of glutamine synthetase and glutamate dehydrogenase in nitrogen assimilation and possibilities for improvement in the nitrogen utilization of crops. J Exp Bot 53:979–987. doi:10.1093/jexbot/53.370.979. 11912240

[22] Steffen-Munsberg F, Vickers C, Kohls H, Land H, Mallin H, Nobili A, Skalden L, van den Bergh T, Joosten H-J, Berglund P, Höhne M, Bornscheuer UT. 2015. Bioinformatic analysis of a PLP-dependent enzyme superfamily suitable for biocatalytic applications. Biotechnol Adv 33:566–604. doi:10.1016/j.biotechadv.2014.12.012. 25575689

[23] Frampton EW, Wood WA. 1961. Carbohydrate oxidation by *Pseudomonas fluorescens*. VI. Conversion of 2-keto-6-phosphogluconate to pyruvate. J Biol Chem 236:2571–2577. doi:10.1016/S0021-9258(19)61700-X. 13894458

[24] Poirel L, Jayol A, Nordmann P. 2017. Polymyxins: antibacterial activity, susceptibility testing, and resistance mechanisms encoded by plasmids or chromosomes. Clin Microbiol Rev 30:557–596. doi:10.1128/CMR.00064-16. 28275006 PMC5355641

[25] Parsons JF, Calabrese K, Eisenstein E, Ladner JE. 2004. Structure of the phenazine biosynthesis enzyme PhzG. Acta Crystallogr D Biol Crystallogr 60:2110–2113. doi:10.1107/S0907444904022474. 15502343

[26] Abdallah II, Quax WJ. 2017. A glimpse into the biosynthesis of terpenoids. KnE Life Sci 3:81–98. doi:10.18502/kls.v3i5.981.

[27] Lievens B, Brouwer M, Vanachter AC, Cammue BP, Thomma BP. 2006. Real-time PCR for detection and quantification of fungal and oomycete tomato pathogens in plant and soil samples. Plant Sci 171:155–165. doi:10.1016/j.plantsci.2006.03.009.

[28] Callahan BJ, McMurdie PJ, Rosen MJ, Han AW, Johnson AJA, Holmes SP. 2016. DADA2: high-resolution sample inference from Illumina amplicon data. Nat Methods 13:581–583. doi:10.1038/nmeth.3869. 27214047 PMC4927377

[29] Murali A, Bhargava A, Wright ES. 2018. IDTAXA: a novel approach for accurate taxonomic classification of microbiome sequences. Microbiome 6:140. doi:10.1186/s40168-018-0521-5. 30092815 PMC6085705

[30] Douglas GM, Maffei VJ, Zaneveld JR, Yurgel SN, Brown JR, Taylor CM, Huttenhower C, Langille MGI. 2020. PICRUSt2 for prediction of metagenome functions. Nat Biotechnol 38:685–688. doi:10.1038/s41587-020-0548-6. 32483366 PMC7365738

[31] Lamichhane JR, Varvaro L. 2013. A new medium for the detection of fluorescent pigment production by pseudomonads. Plant Pathol 62:624–632. doi:10.1111/j.1365-3059.2012.02670.x.

