# Supplementary material for: Transition from Ginseng Root Rot Disease-Conducive Soil to -Suppressive Soil Mediated by Pseudomonadaceae
Source: Microbiol Spectr. 2023 Jul 5;11(4):e01150-23. doi: 10.1128/spectrum.01150-23 (PMC10433981; doi:10.1128/spectrum.01150-23)
Supplement: Supplemental file 1 — Supplemental material. Download spectrum.01150-23-s0001.docx, DOCX file, 1.7 MB [file spectrum.01150-23-s0001.docx]

**Supplementary Information**

*for*

**Transition from Ginseng Root Disease Conducive Soil to Suppressive Soil through Pseudomonadaceae**

Gyeongjun Cho^1^, Da-Ran Kim^2^, and Youn-Sig Kwak^1,2^*

This file includes 3 tables and 9 figures.

**Table S1.** 16S rRNA sequences read quality control results in this study

| Sample | Raw forward | Raw reverse | Filtered forward | Filtered reverse | Denoised forward | Denoised reverse | Merged reads | Removed chimera | Only bacteria |
| --- | --- | --- | --- | --- | --- | --- | --- | --- | --- |
| Bulk soil1 | 135,740 | 135,740 | 109,020 | 109,020 | 107,409 | 107,277 | 102,948 | 101,896 | 77,710 |
| Bulk soil3 | 109,118 | 109,118 | 89,284 | 89,284 | 87,342 | 87,661 | 80,443 | 79,476 | 60,380 |
| Bulk soil5 | 108,373 | 108,373 | 87,635 | 87,635 | 85,880 | 85,944 | 81,637 | 80,849 | 61,782 |
| Bulk soil7 | 117,902 | 117,902 | 95,437 | 95,437 | 93,654 | 93,732 | 88,656 | 87,617 | 65,011 |
| Con1 | 135,293 | 135,293 | 112,407 | 112,407 | 110,807 | 111,082 | 107,704 | 100,410 | 88,232 |
| Con2 | 120,437 | 120,437 | 101,712 | 101,712 | 100,242 | 100,586 | 97,643 | 94,145 | 83,265 |
| Con3 | 116,577 | 116,577 | 98,557 | 98,557 | 97,298 | 97,570 | 95,055 | 91,920 | 85,353 |
| Con4 | 127,187 | 127,187 | 107,970 | 107,970 | 106,570 | 106,861 | 103,652 | 97,890 | 87,207 |
| Con5 | 117,174 | 117,174 | 98,952 | 98,952 | 97,139 | 97,577 | 94,478 | 92,613 | 81,272 |
| Asn1 | 130,668 | 130,668 | 109,362 | 109,362 | 107,578 | 107,791 | 103,861 | 99,761 | 88,339 |
| Asn2 | 142,098 | 142,098 | 118,429 | 118,429 | 116,800 | 117,098 | 113,642 | 108,636 | 94,079 |
| Asn3 | 137,985 | 137,985 | 115,821 | 115,821 | 114,094 | 114,287 | 110,825 | 108,050 | 99,084 |
| Asn4 | 130,545 | 130,545 | 111,032 | 111,032 | 109,532 | 109,805 | 106,888 | 103,664 | 87,487 |
| Asn5 | 135,007 | 135,007 | 113,546 | 113,546 | 111,783 | 112,359 | 108,730 | 105,872 | 96,785 |
| Asp1 | 124,729 | 124,729 | 104,635 | 104,635 | 103,624 | 103,534 | 100,812 | 98,097 | 93,081 |
| Asp2 | 120,999 | 120,999 | 101,242 | 101,242 | 99,880 | 100,087 | 97,014 | 93,474 | 81,978 |
| Asp3 | 127,492 | 127,492 | 108,500 | 108,500 | 107,012 | 107,422 | 103,985 | 98,544 | 82,075 |
| Asp4 | 108,186 | 108,186 | 90,231 | 90,231 | 89,225 | 89,453 | 87,395 | 85,076 | 77,795 |
| Asp5 | 131,119 | 131,119 | 111,391 | 111,391 | 109,821 | 110,025 | 105,977 | 102,663 | 86,550 |
| Glu1 | 124,255 | 124,255 | 100,005 | 100,005 | 97,994 | 98,155 | 93,410 | 87,059 | 77,474 |
| Glu2 | 121,883 | 121,883 | 103,666 | 103,666 | 102,555 | 102,749 | 100,507 | 98,389 | 96,369 |
| Glu3 | 123,884 | 123,884 | 103,052 | 103,052 | 101,688 | 101,870 | 98,303 | 93,768 | 82,126 |
| Glu4 | 121,798 | 121,798 | 103,700 | 103,700 | 102,247 | 102,499 | 99,305 | 96,418 | 87,391 |
| Glu5 | 129,129 | 129,129 | 110,380 | 110,380 | 108,724 | 108,942 | 104,912 | 100,771 | 92,840 |
| Val1 | 130,554 | 130,554 | 109,081 | 109,081 | 107,553 | 107,821 | 104,303 | 99,060 | 90,504 |
| Val2 | 127,937 | 127,937 | 106,993 | 106,993 | 105,425 | 105,702 | 102,545 | 99,579 | 85,152 |
| Val3 | 141,858 | 141,858 | 118,938 | 118,938 | 116,710 | 117,132 | 111,535 | 102,631 | 94,505 |
| Val4 | 145,908 | 145,908 | 122,285 | 122,285 | 120,778 | 121,183 | 118,096 | 115,602 | 106,148 |
| Val5 | 138,014 | 138,014 | 117,009 | 117,009 | 115,737 | 116,006 | 113,633 | 111,167 | 103,200 |
| NH4Cl1 | 131,476 | 131,476 | 108,628 | 108,628 | 106,881 | 107,500 | 104,035 | 102,510 | 91,986 |
| NH4Cl2 | 119,960 | 119,960 | 100,787 | 100,787 | 99,183 | 99,640 | 96,718 | 95,520 | 82,161 |
| NH4Cl3 | 141,340 | 141,340 | 119,100 | 119,100 | 117,368 | 117,637 | 114,037 | 110,325 | 90,480 |
| NH4Cl4 | 125,408 | 125,408 | 107,027 | 107,027 | 105,260 | 105,596 | 101,995 | 99,000 | 85,947 |
| NH4Cl5 | 103,980 | 103,980 | 79,937 | 79,937 | 78,569 | 78,804 | 76,152 | 75,470 | 65,627 |

**Table S2.** Disease index or *F. solani* negative correlation pathways and description

| MetaCyc pathway ID | Pathway description | KEGG Onthology ID | KEGG Onthology ID description in PICRUSt2 |
| --- | --- | --- | --- |
| AST-PWY | L-arginine degradation II (AST pathway) | K00673 | astA; arginine N-succinyltransferase [EC:2.3.1.109] |
|  |  | K01484 | astB; succinylarginine dihydrolase [EC:3.5.3.23] |
|  |  | K00840 | astC; succinylornithine aminotransferase [EC:2.6.1.81] |
|  |  | K06447 | astD; succinylglutamic semialdehyde dehydrogenase [EC:1.2.1.71] |
|  |  | K05526 | astE; succinylglutamate desuccinylase [EC:3.5.1.96] |
| ARGORNPROST-PWY | L-arginine degradation (Stickland reaction) | K01478 | arcA; arginine deiminase [EC:3.5.3.6] |
|  |  | K00611 | OTC, argF, argI; ornithine carbamoyltransferase [EC:2.1.3.3] |
|  |  | K00926 | arcC; carbamate kinase [EC:2.7.2.2] |
|  |  | K21898 | not included PICRUSt2 (version 2.3.0-b) |
|  |  | K17899 | oraS; D-ornithine 4,5-aminomutase subunit alpha [EC:5.4.3.5] |
|  |  | K21672 | not included PICRUSt2 (version 2.3.0-b) |
|  |  | K21399 | not included PICRUSt2 (version 2.3.0-b) |
|  |  | K21400 | not included PICRUSt2 (version 2.3.0-b) |
|  |  | K00819 | rocD, OAT; ornithine--oxo-acid transaminase [EC:2.6.1.13] |
|  |  | K00286 | proC; pyrroline-5-carboxylate reductase [EC:1.5.1.2] |
|  |  | K01777 | prdF; proline racemase [EC:5.1.1.4] |
|  |  | K10793 | prdA; D-proline reductase (dithiol) PrdA [EC:1.21.4.1] |
|  |  | K10794 | prdB; D-proline reductase (dithiol) PrdB [EC:1.21.4.1] |
|  |  | K01750 | E4.3.1.12, ocd; ornithine cyclodeaminase [EC:4.3.1.12] |
| DHGLUCONATE-PYR-CAT-PWY | Glucose degradation (oxidative) | K00117 | gcd; quinoprotein glucose dehydrogenase [EC:1.1.5.2] |
|  |  | K01053 | E3.1.1.17, gnl, RGN; gluconolactonase [EC:3.1.1.17] |
|  |  | K06151 | E1.1.99.3A; gluconate 2-dehydrogenase alpha chain [EC:1.1.99.3] |
|  |  | K06152 | E1.1.99.3G; gluconate 2-dehydrogenase gamma chain [EC:1.1.99.3] |
|  |  | K11441 | kguK; dehydrogluconokinase [EC:2.7.1.13] |
|  |  | K00032 | E1.1.1.43; phosphogluconate 2-dehydrogenase [EC:1.1.1.43] |
| PWY0_1338 | Polymyxin resistance | K10011 | arnA, pmrI; UDP-4-amino-4-deoxy-L-arabinose formyltransferase / UDP-glucuronic acid dehydrogenase (UDP-4-keto-hexauronic acid decarboxylating) [EC:2.1.2.13, 1.1.1.305] |
|  |  | K07806 | arnB, pmrH; UDP-4-amino-4-deoxy-L-arabinose-oxoglutarate aminotransferase [EC:2.6.1.87] |
|  |  | K15895 | not included PICRUSt2 (version 2.3.0-b) |
|  |  | K10011 | arnA, pmrI; UDP-4-amino-4-deoxy-L-arabinose formyltransferase / UDP-glucuronic acid dehydrogenase (UDP-4-keto-hexauronic acid decarboxylating) [EC:2.1.2.13, 1.1.1.305] |
|  |  | K10012 | arnC, pmrF; undecaprenyl-phosphate 4-deoxy-4-formamido-L-arabinose transferase [EC:2.4.2.53] |
|  |  | K07264 | arnT, pmrK; 4-amino-4-deoxy-L-arabinose transferase [EC:2.4.2.43] |
| PYRIDOXSYN-PWY | pyridoxal 5'-phosphate biosynthesis I | K03472 | epd; D-erythrose 4-phosphate dehydrogenase [EC:1.2.1.72] |
|  |  | K03473 | pdxB; erythronate-4-phosphate dehydrogenase [EC:1.1.1.290] |
|  |  | K00831 | serC, PSAT1; phosphoserine aminotransferase [EC:2.6.1.52] |
|  |  | K00097 | pdxA; 4-hydroxythreonine-4-phosphate dehydrogenase [EC:1.1.1.262] |
|  |  | K01662 | dxs; 1-deoxy-D-xylulose-5-phosphate synthase [EC:2.2.1.7] |
|  |  | K03474 | pdxJ; pyridoxine 5-phosphate synthase [EC:2.6.99.2] |
|  |  | K00275 | pdxH, PNPO; pyridoxamine 5'-phosphate oxidase [EC:1.4.3.5] |
| PWY-5910 | Superpathway of geranylgeranyldiphosphate biosynthesis I (via mevalonate) | K00626 | E2.3.1.9, atoB; acetyl-CoA C-acetyltransferase [EC:2.3.1.9] |
|  |  | K00632 | fadA, fadI; acetyl-CoA acyltransferase [EC:2.3.1.16] |
|  |  | K07508 | ACAA2; acetyl-CoA acyltransferase 2 [EC:2.3.1.16] |
|  |  | K07509 | not included PICRUSt2 (version 2.3.0-b) |
|  |  | K07513 | not included PICRUSt2 (version 2.3.0-b) |
|  |  | K01641 | E2.3.3.10; hydroxymethylglutaryl-CoA synthase [EC:2.3.3.10] |
|  |  | K00021 | HMGCR; hydroxymethylglutaryl-CoA reductase (NADPH) [EC:1.1.1.34] |
|  |  | K00869 | E2.7.1.36, MVK, mvaK1; mevalonate kinase [EC:2.7.1.36] |
|  |  | K00938 | E2.7.4.2, mvaK2; phosphomevalonate kinase [EC:2.7.4.2] |
|  |  | K13273 | not included PICRUSt2 (version 2.3.0-b) |
|  |  | K01597 | MVD, mvaD; diphosphomevalonate decarboxylase [EC:4.1.1.33] |
|  |  | K01823 | idi, IDI; isopentenyl-diphosphate Delta-isomerase [EC:5.3.3.2] |
|  |  | K12742 | not included PICRUSt2 (version 2.3.0-b) |
| PWY-7391 | Isoprene biosynthesis II (engineered) | K01662 | dxs; 1-deoxy-D-xylulose-5-phosphate synthase [EC:2.2.1.7] |
|  |  | K00099 | dxr; 1-deoxy-D-xylulose-5-phosphate reductoisomerase [EC:1.1.1.267] |
|  |  | K00991 | ispD; 2-C-methyl-D-erythritol 4-phosphate cytidylyltransferase [EC:2.7.7.60] |
|  |  | K12506 | ispDF; 2-C-methyl-D-erythritol 4-phosphate cytidylyltransferase / 2-C-methyl-D-erythritol 2,4-cyclodiphosphate synthase [EC:2.7.7.60, 4.6.1.12] |
|  |  | K00919 | ispE; 4-diphosphocytidyl-2-C-methyl-D-erythritol kinase [EC:2.7.1.148] |
|  |  | K01770 | ispF; 2-C-methyl-D-erythritol 2,4-cyclodiphosphate synthase [EC:4.6.1.12] |
|  |  | K12506 | ispDF; 2-C-methyl-D-erythritol 4-phosphate cytidylyltransferase / 2-C-methyl-D-erythritol 2,4-cyclodiphosphate synthase [EC:2.7.7.60, 4.6.1.12] |
|  |  | K03526 | gcpE, ispG; (E)-4-hydroxy-3-methylbut-2-enyl-diphosphate synthase [EC:1.17.7.1, 1.17.7.3] |
|  |  | K03527 | ispH, lytB; 4-hydroxy-3-methylbut-2-en-1-yl diphosphate reductase [EC:1.17.7.4] |
|  |  | K01823 | idi, IDI; isopentenyl-diphosphate Delta-isomerase [EC:5.3.3.2] |
|  |  | K13787 | idsA; geranylgeranyl diphosphate synthase, type I [EC:2.5.1.1, 2.5.1.10, 2.5.1.29] |
| PWY-922 | Mevalonate pathway I (eukaryotes and bacteria) | K00626 | E2.3.1.9, atoB; acetyl-CoA C-acetyltransferase [EC:2.3.1.9] |
|  |  | K00632 | fadA, fadI; acetyl-CoA acyltransferase [EC:2.3.1.16] |
|  |  | K07508 | ACAA2; acetyl-CoA acyltransferase 2 [EC:2.3.1.16] |
|  |  | K07509 | not included PICRUSt2 (version 2.3.0-b) |
|  |  | K07513 | not included PICRUSt2 (version 2.3.0-b) |
|  |  | K01641 | E2.3.3.10; hydroxymethylglutaryl-CoA synthase [EC:2.3.3.10] |
|  |  | K00021 | HMGCR; hydroxymethylglutaryl-CoA reductase (NADPH) [EC:1.1.1.34] |
|  |  | K00869 | E2.7.1.36, MVK, mvaK1; mevalonate kinase [EC:2.7.1.36] |
|  |  | K00938 | E2.7.4.2, mvaK2; phosphomevalonate kinase [EC:2.7.4.2] |
|  |  | K13273 | not included PICRUSt2 (version 2.3.0-b) |
|  |  | K01597 | MVD, mvaD; diphosphomevalonate decarboxylase [EC:4.1.1.33] |
|  |  | K01823 | idi, IDI; isopentenyl-diphosphate Delta-isomerase [EC:5.3.3.2] |

**Table S3.** Information on identifying bacteria with at least one gene in each of the seven important pathways

| ASV ID | Domain | Phylum | Class | Order | Family | Genus |
| --- | --- | --- | --- | --- | --- | --- |
| ASV_1 | Bacteria | Proteobacteria | Gammaproteobacteria | Pseudomonadales | Pseudomonadaceae | Pseudomonas |
| ASV_1238 | Bacteria | Sumerlaeota | Sumerlaeia | Sumerlaeales | Sumerlaeaceae | Sumerlaea |
| ASV_1354 | Bacteria | Proteobacteria | Gammaproteobacteria | Burkholderiales | Oxalobacteraceae | Massilia |
| ASV_14 | Bacteria | Proteobacteria | Gammaproteobacteria | Pseudomonadales | Pseudomonadaceae | Pseudomonas |
| ASV_1494 | Bacteria | Proteobacteria | Gammaproteobacteria | Enterobacterales | Enterobacteriaceae |  |
| ASV_160 | Bacteria | Proteobacteria | Gammaproteobacteria | Pseudomonadales | Pseudomonadaceae | Pseudomonas |
| ASV_17 | Bacteria | Proteobacteria | Gammaproteobacteria | Pseudomonadales | Pseudomonadaceae | Pseudomonas |
| ASV_1800 | Bacteria | Myxococcota | Myxococcia | Myxococcales | Myxococcaceae |  |
| ASV_195 | Bacteria | Proteobacteria | Gammaproteobacteria | Pseudomonadales | Pseudomonadaceae |  |
| ASV_20 | Bacteria | Proteobacteria | Gammaproteobacteria | Enterobacterales | Enterobacteriaceae |  |
| ASV_21 | Bacteria | Proteobacteria | Gammaproteobacteria | Enterobacterales | Enterobacteriaceae |  |
| ASV_212 | Bacteria | Proteobacteria | Gammaproteobacteria | Burkholderiales | Oxalobacteraceae |  |
| ASV_23 | Bacteria | Proteobacteria | Gammaproteobacteria | Pseudomonadales | Pseudomonadaceae | Pseudomonas |
| ASV_28 | Bacteria | Proteobacteria | Gammaproteobacteria | Pseudomonadales | Pseudomonadaceae | Pseudomonas |
| ASV_293 | Bacteria | Proteobacteria | Gammaproteobacteria | Burkholderiales | Burkholderiaceae | Burkholderia-Caballeronia-Paraburkholderia |
| ASV_3 | Bacteria | Proteobacteria | Gammaproteobacteria | Pseudomonadales | Pseudomonadaceae | Pseudomonas |
| ASV_348 | Bacteria | Proteobacteria | Gammaproteobacteria | Burkholderiales | Burkholderiaceae |  |
| ASV_361 | Bacteria | Proteobacteria | Gammaproteobacteria | Burkholderiales | Burkholderiaceae | Burkholderia-Caballeronia-Paraburkholderia |
| ASV_400 | Bacteria | Proteobacteria | Gammaproteobacteria | Pseudomonadales | Pseudomonadaceae | Pseudomonas |
| ASV_402 | Bacteria | Proteobacteria | Gammaproteobacteria | Burkholderiales | Burkholderiaceae |  |
| ASV_419 | Bacteria | Proteobacteria | Gammaproteobacteria | Burkholderiales | Burkholderiaceae | Burkholderia-Caballeronia-Paraburkholderia |
| ASV_445 | Bacteria | Proteobacteria | Gammaproteobacteria | Pseudomonadales | Pseudomonadaceae | Pseudomonas |
| ASV_486 | Bacteria | Proteobacteria | Gammaproteobacteria | Burkholderiales | Burkholderiaceae | Burkholderia-Caballeronia-Paraburkholderia |
| ASV_490 | Bacteria | Myxococcota | Myxococcia | Myxococcales | Myxococcaceae | Myxococcus |
| ASV_506 | Bacteria | Proteobacteria | Gammaproteobacteria | Burkholderiales | Burkholderiaceae | Cupriavidus |
| ASV_53 | Bacteria | Proteobacteria | Gammaproteobacteria | Enterobacterales | Enterobacteriaceae |  |
| ASV_563 | Bacteria | Proteobacteria | Gammaproteobacteria | Pseudomonadales | Pseudomonadaceae | Pseudomonas |
| ASV_694 | Bacteria | Proteobacteria | Gammaproteobacteria | Pseudomonadales | Pseudomonadaceae |  |
| ASV_707 | Bacteria | Proteobacteria | Gammaproteobacteria | Burkholderiales | Oxalobacteraceae |  |
| ASV_859 | Bacteria | Proteobacteria | Gammaproteobacteria | Enterobacterales | Enterobacteriaceae |  |
| ASV_86 | Bacteria | Proteobacteria | Gammaproteobacteria | Pseudomonadales | Pseudomonadaceae | Pseudomonas |
| ASV_94 | Bacteria | Proteobacteria | Gammaproteobacteria | Burkholderiales | Oxalobacteraceae | Massilia |


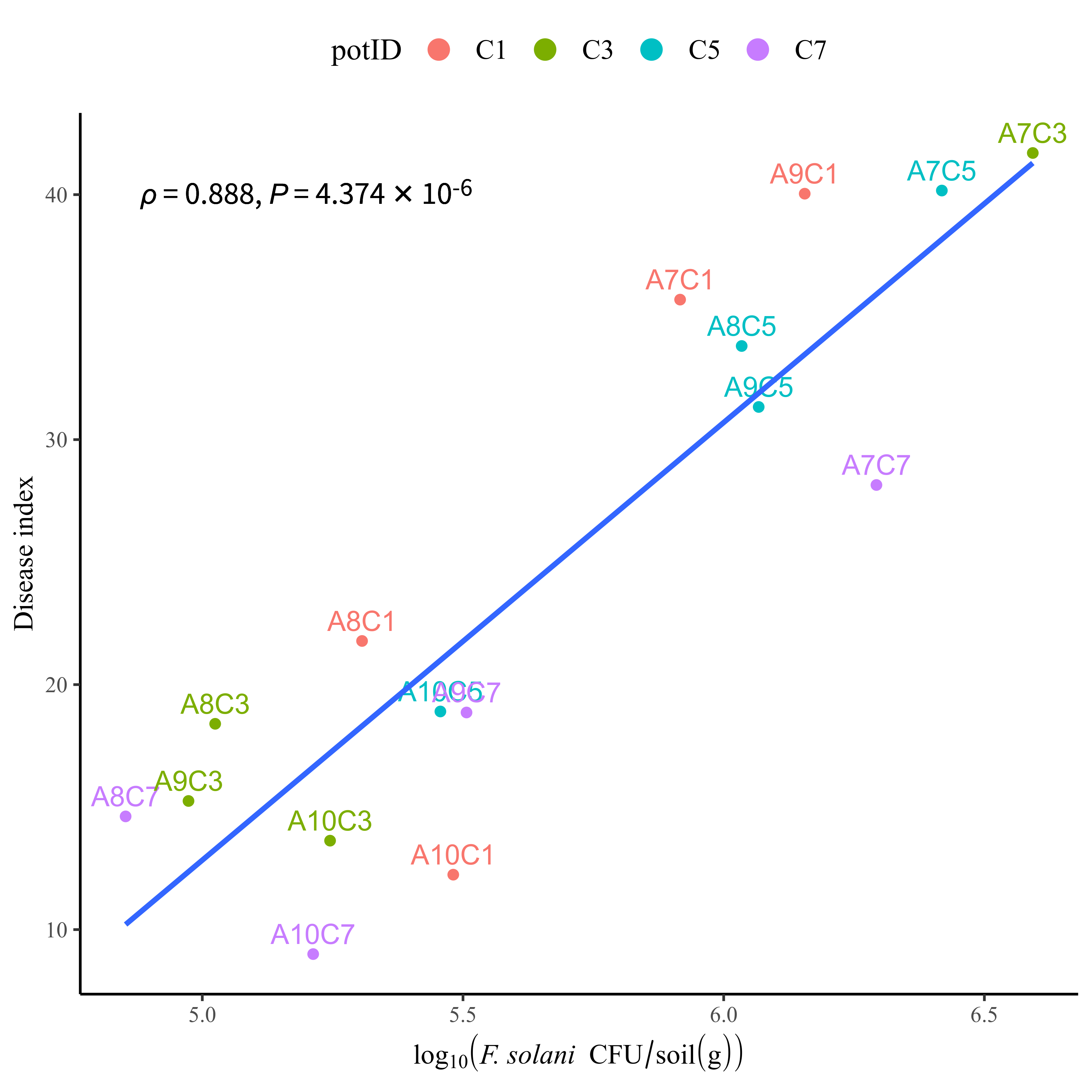


**Fig** **S1.** Correlation between *Fusarium solani* concentration and disease index during the last planting cycle (7th to 10th) in the previous study (19). Pearson's correlation analysis was performed to assess the association between *F. solani* density and root rot disease index. A significant positive correlation was observed between the density of *F. solani* and the disease index.


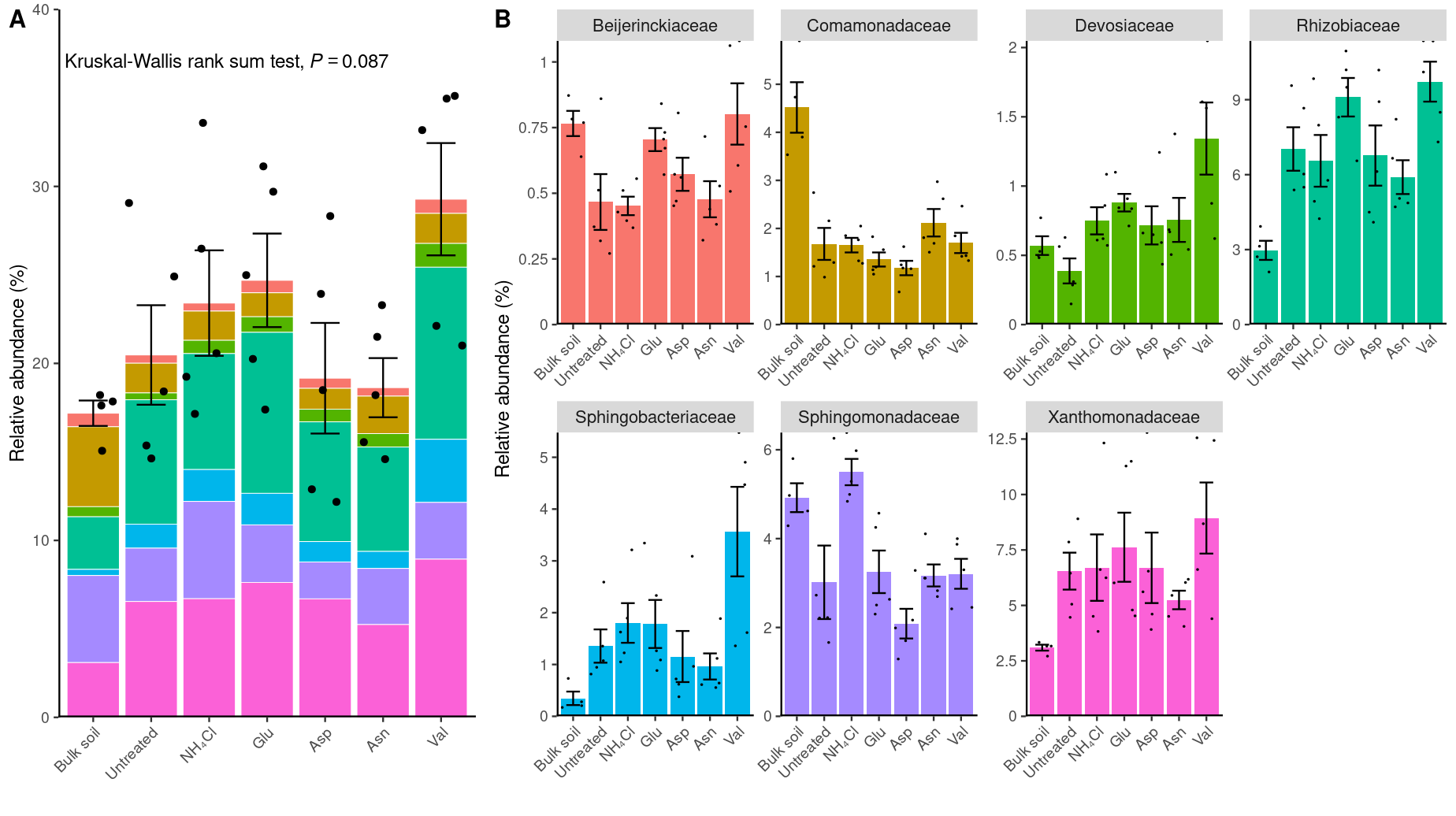


**Fig S2.** Relative abundance of the influence taxa to reduce the root rot disease, as identified in a previous study. The selected influence taxa were assessed for their potential to reduce root rot disease. **A** The overall relative abundance of the initial influencer taxa is represented by a thick bar with filled colors indicating each family. The vertical segment represents the mean ± standard error. No significant differences were observed between nitrogen treatments (Kruskal-Wallis sum rank test, *P* < 0.05). **B** Detailed information on the relative abundance of each bacterial family is presented.


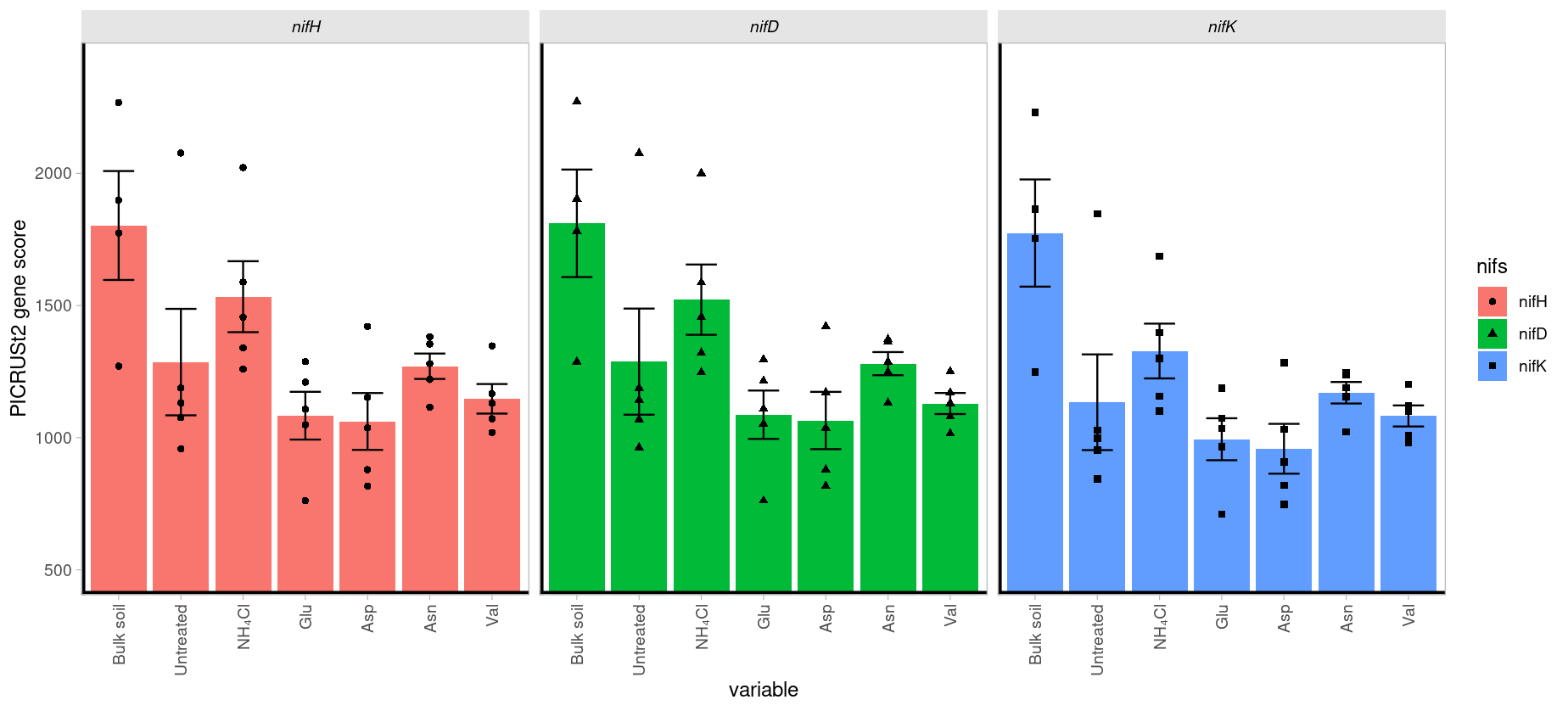


**Fig S3.** PICRUSt2 *nifHDK* gene scores for a pot. The pot score is the sum of scores for each ASV present in the pot. The bar represents the average of the sum scores. Prior to the summation, individual scores for each ASV were used to calculate the GOO index.


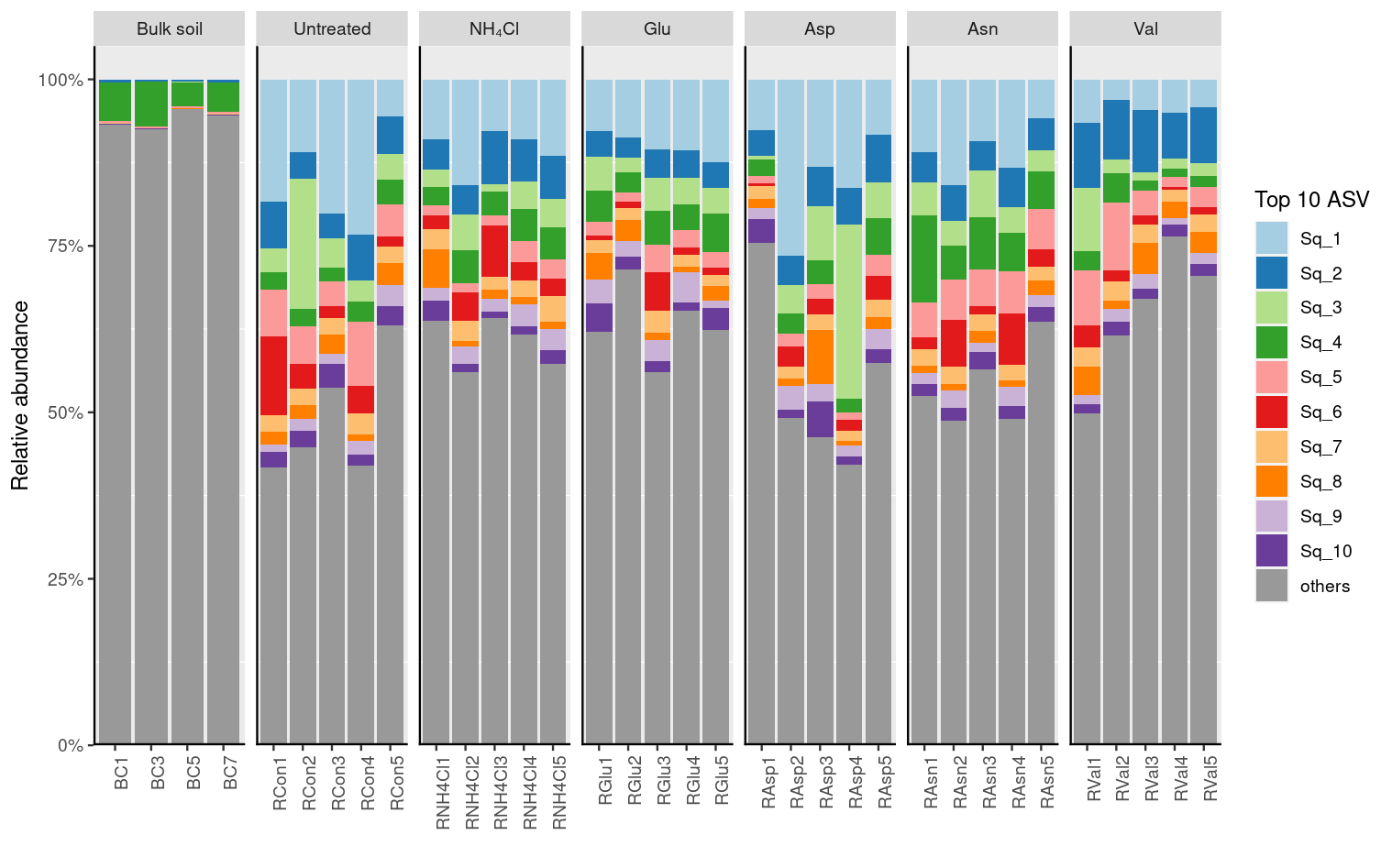


**Fig S4.** Relative abundance at ASV level. The top 10 ASVs are represented by bar color.


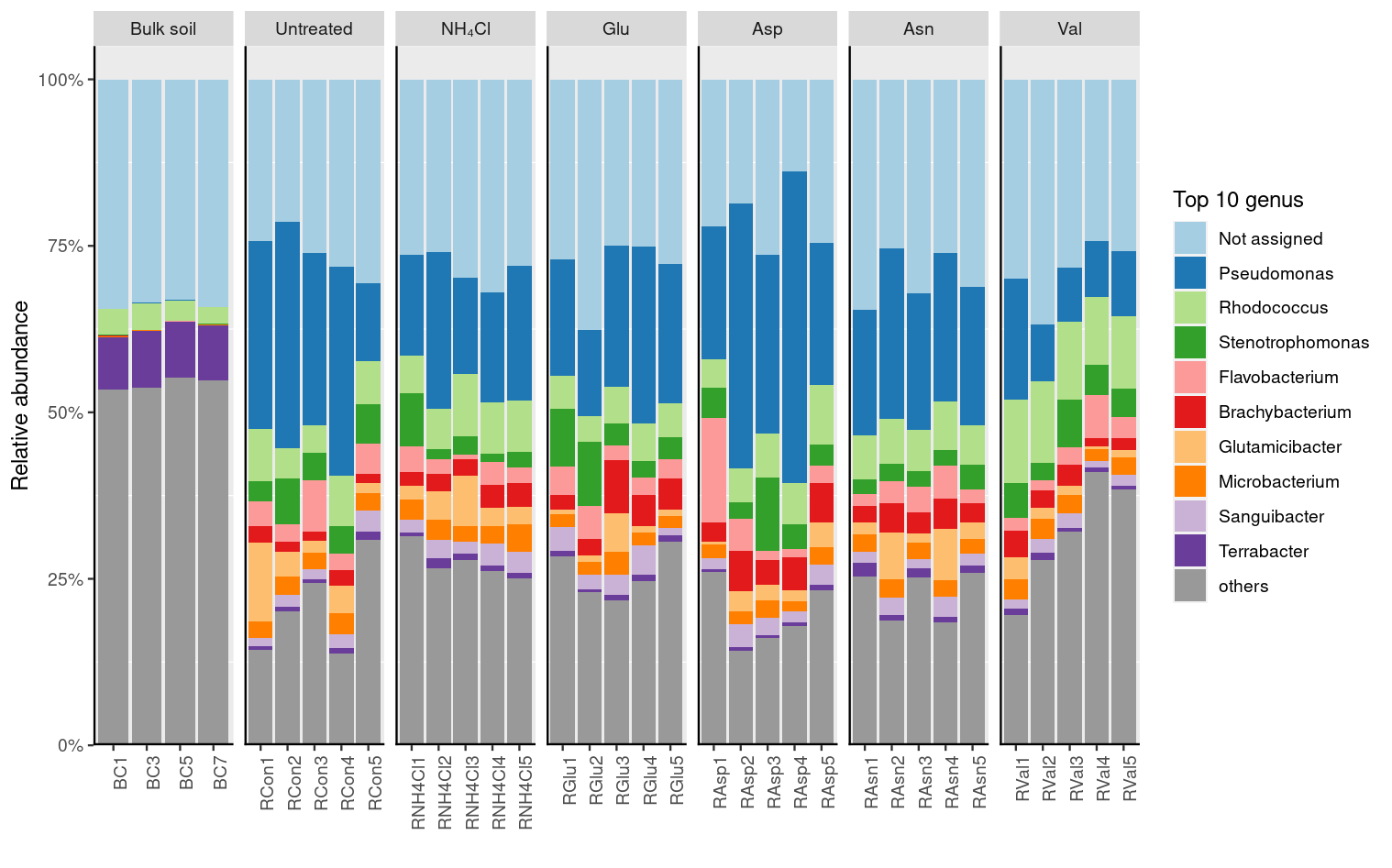


**Fig S5.** Relative abundance at the genus level. the top 10 genus groups are represented by bar color.


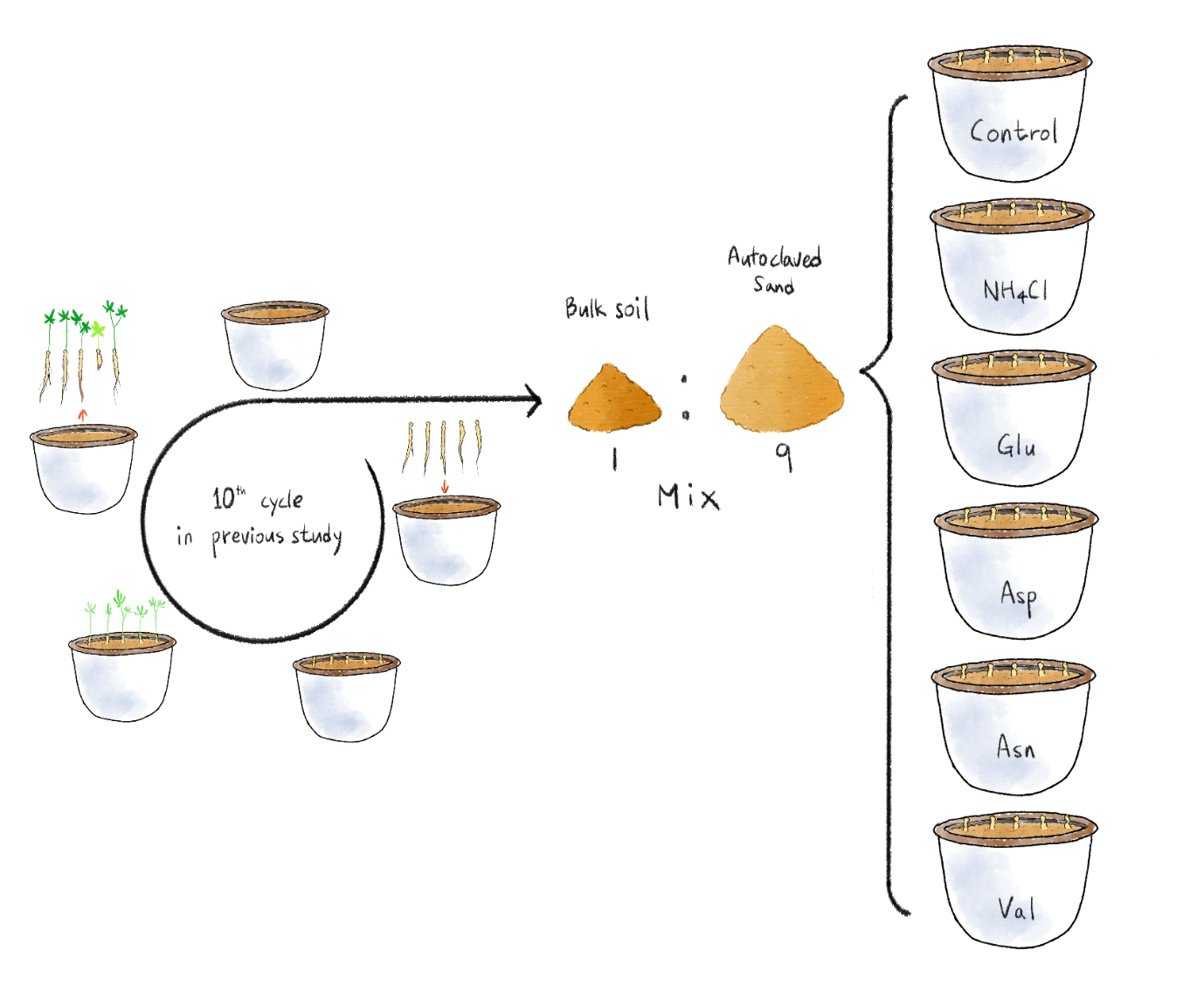


**Fig S6**. Schematic representation of the nitrogen treatment blueprint. The experimental setup includes a control group with two samples: bulk soil prior to ginseng planting and the rhizosphere of ginseng root left untreated for 20 days after planting. The figure illustrates the division of the control group sample.


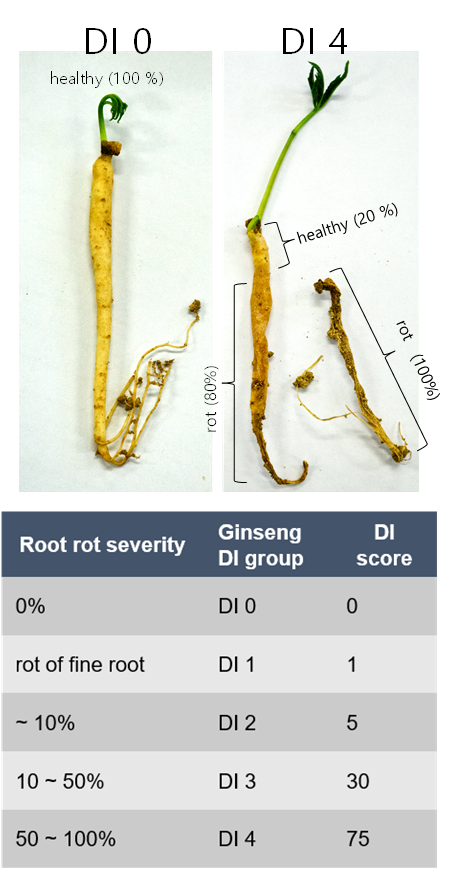


**Fig S7.** Description of disease index. disease index (DI) is represented by the middle value of the severity percentage.


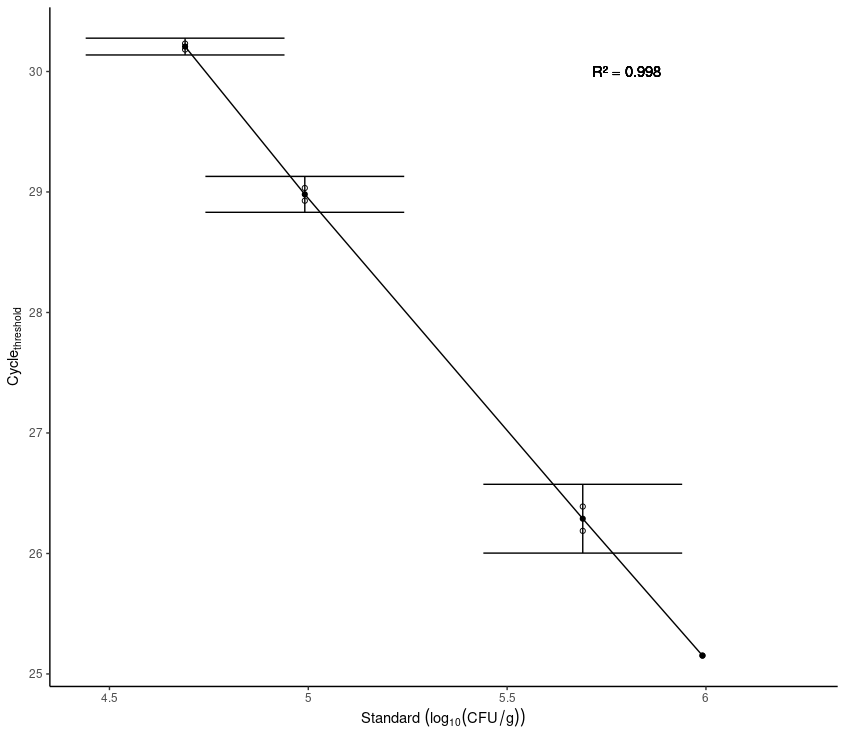


**Fig S8.** The standard segment between qPCR threshold cycle and *F. solani* density.


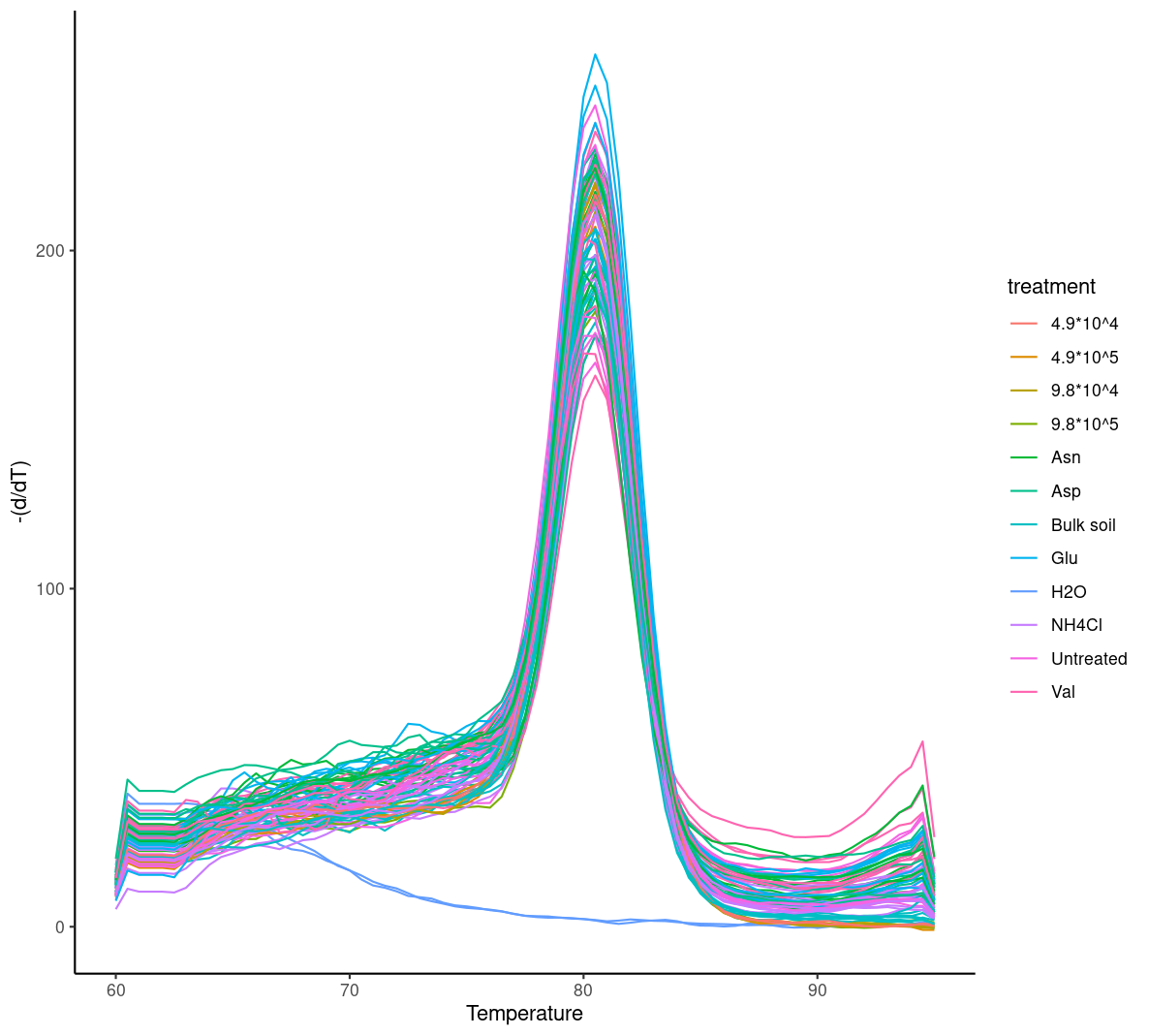


**Fig S9**. Melting curve analysis of qPCR for *Fusarium solani* density measurement. The legend indicates the standard samples with known *F. solani* colony forming units (CFU). The y-axis shows the change in SYBR green signal over time. The appearance of a peak at 80°C for each sample confirms the reliable detection of *F. solani* by qPCR.
